# Tiled material systems: Exploring biodiversity and multifunctionality of a universal and structural motif

**DOI:** 10.1093/pnasnexus/pgaf046

**Published:** 2025-11-11

**Authors:** Jana Ciecierska-Holmes, Nikolai Rosenthal, Jan Wölfer, Felix Rasehorn, Binru Yang, Mai-Lee Van Le, Lennart Eigen, John A Nyakatura, Mason N Dean

**Affiliations:** Cluster of Excellence ‘Matters of Activity: Image Space Material’, Humboldt-Universität zu Berlin, Unter den Linden 6, 10099 Berlin, Germany; Max Planck Institute of Colloids and Interfaces, Potsdam Science Park, Am Mühlenberg 1, 14476 Potsdam, Germany; Institute of Biology, Humboldt-Universität zu Berlin, Invalidenstraße 42, 10115 Berlin, Germany; Cluster of Excellence ‘Matters of Activity: Image Space Material’, Humboldt-Universität zu Berlin, Unter den Linden 6, 10099 Berlin, Germany; Max Planck Institute of Colloids and Interfaces, Potsdam Science Park, Am Mühlenberg 1, 14476 Potsdam, Germany; Institute of Biology, Humboldt-Universität zu Berlin, Invalidenstraße 42, 10115 Berlin, Germany; Cluster of Excellence ‘Matters of Activity: Image Space Material’, Humboldt-Universität zu Berlin, Unter den Linden 6, 10099 Berlin, Germany; Department of Product Design, Weißensee Kunsthochschule Berlin, Bühringstraße 20, 13086 Berlin, Germany; Cluster of Excellence ‘Matters of Activity: Image Space Material’, Humboldt-Universität zu Berlin, Unter den Linden 6, 10099 Berlin, Germany; Max Planck Institute of Colloids and Interfaces, Potsdam Science Park, Am Mühlenberg 1, 14476 Potsdam, Germany; Cluster of Excellence ‘Matters of Activity: Image Space Material’, Humboldt-Universität zu Berlin, Unter den Linden 6, 10099 Berlin, Germany; Institute of Biology, Humboldt-Universität zu Berlin, Invalidenstraße 42, 10115 Berlin, Germany; Cluster of Excellence ‘Matters of Activity: Image Space Material’, Humboldt-Universität zu Berlin, Unter den Linden 6, 10099 Berlin, Germany; Institute of Biology, Humboldt-Universität zu Berlin, Invalidenstraße 42, 10115 Berlin, Germany; Bernstein Center for Computational Neuroscience Berlin, Humboldt-Universität zu Berlin, Unter den Linden 6, 10099 Berlin, Germany; Cluster of Excellence ‘Matters of Activity: Image Space Material’, Humboldt-Universität zu Berlin, Unter den Linden 6, 10099 Berlin, Germany; Institute of Biology, Humboldt-Universität zu Berlin, Invalidenstraße 42, 10115 Berlin, Germany; Cluster of Excellence ‘Matters of Activity: Image Space Material’, Humboldt-Universität zu Berlin, Unter den Linden 6, 10099 Berlin, Germany; Max Planck Institute of Colloids and Interfaces, Potsdam Science Park, Am Mühlenberg 1, 14476 Potsdam, Germany; Department of Infectious Disease & Public Health, City University of Hong Kong, 1A-501, Block 1, To Yuen Building, 31 To Yuen Street, Tat Chee Avenue, Kowloon, Hong Kong; Centre for Nature-Inspired Engineering, City University of Hong Kong, G7410, 7/F, Green Zone, Yeung Kin Man Academic Building, Tat Chee Avenue, Kowloon, Hong Kong

**Keywords:** material design, biological architecture, biomimetics, composite biomaterials, multivariable classification

## Abstract

Humans are drawn to patterns and hierarchies in nature, mimicking them particularly in decoration and architecture. Natural patterns, however, are never purely esthetic and, since evolution works on a variety of factors simultaneously, natural structural systems are intrinsically multifunctional. In order to understand the roles that structural patterns play in biology (and therefore their potential capabilities and utilization in design, architecture and engineering), we need to catalog and encapsulate the diversity of examples and the materials involved. Here, we provide a first classification of biological “tilings,” tessellated natural architectures that involve the repeated pattern of geometric, discrete elements bound by a joint material. By examining 100 examples across the Tree of Life, we reveal this natural structural motif is unexpectedly prevalent: we cover a huge taxonomic diversity, eight orders of magnitude in size scale, and myriad morphologies and functions ranging from optics to armor, allowing us to construct a hierarchical system of eight variables to classify form, function, and materiality in biological tilings. Using diverse means of data analysis (including multiple correspondence analysis), we show this database can be explored to reveal fundamental links among anatomical characteristics and functions as well as connections among and within taxonomic groups. Our resulting collection of “tessellated materials” and its companion website act therefore as a multidisciplinary meeting point (e.g. for biologists, designers, engineers, architects). In this way, our database offers windows for exploring selective pressures and trade-offs and a launchpad for future research and collaborative, cross-disciplinary, bioinspired projects.

Significance Statement Humans are drawn to natural patterns, mimicking them for both esthetics and functionality. Biological tessellated patterns or “tilings,” for example, are abundant and diverse; however, research around them is limited to a handful of popular examples, many actually cellular foams rather than tilings. Here, we provide a practical classification of biological tilings, examining a huge taxonomic diversity from across the Tree of Life to classify form, function, and materiality for the myriad observed morphologies. Through multivariate analyses, we reveal cross-cutting architectural themes across diverse taxonomic groups, demonstrating the database's strength in revealing fundamental links among structural characteristics and function. With its companion website, our collection and classification system is therefore a multidisciplinary meeting point for biodiversity, biomaterials, and bioinspired projects.

## Introduction

Biological architectures are often hierarchical, constructed from repeating patterns of smaller components (1–5). Humans have been drawn to natural patterns since antiquity and our conceptualizations remain especially dominated by particular forms (6–10). Designers and architects, for instance, are fascinated by spirals (e.g. of fern fiddleheads, nautilus shells, reptile tails) and “cellular foams,” arrays of compartmentalized closed- or open-celled voids (e.g. of honeycombs, wood, and cancellous bone) (10–12). Cellular foams are prized in architecture and mechanical engineering for their combination of esthetics, high strength, and low weight (13). However, these architectures often are not especially dynamic: although adjacent cells share walls, forming a homogeneous system that evenly distributes load, their structure prevents shifting, except when buckling under load (14, 15).

Tessellations are effectively the structural inverse of cellular foams, comprising tilings of solid subunits rather than arrays of compartments (16–18), with an in-built potential for activity: the subunits being distinct from each other permits an inherent flexibility, allowing individual tiles to be displaced relative to one another or even replaced (19–22). For these reasons, tessellations have been standard motifs for manmade armors, tiled roofings, and pavements (18, 23). These clearly echo (and were often inspired by) the imbricate scalings of fishes and reptiles, but represent just one type of structural tiling (4). In contrast to natural cellular foams, tessellations in nature are far less discussed, especially as many examples are microscopic, concealed, or less familiar.

Here, we present a collection and characterization of biological tilings, exploring the prevalence and functions of this structural patterning across the Tree of Life. Human efforts at biomimicry have often led to myopic pursuits of natural “optimization” of single performance traits (e.g. strong adhesion, high stiffness, iridescence, superhydrophobicity) (24). In this way, we can lose sight of what makes biology inspiring: the organism and its natural context, the complex and diverse interactions of physiology, evolutionary history, and environment. Our project takes an inverted approach to material discovery. Rather than working from a particular taxonomic group or function, we look for tessellated structural patterns without those constraints, using collected examples to build and refine a classification system. Drawing on more than 100 biological examples, from viruses to vertebrates and over eight orders of magnitude in size scale, we construct a multivariable system of classification and build an accompanying interactive website (https://tessellated-materials.mpikg.mpg.de/) for what turns out to be a surprisingly common but largely unexamined trope in biological architecture. Instead of drilling deeply into specific organismal examples, we use the collection to capture broader trends and underline cross-cutting themes in the evolution of biological, structural patterns. In this way, we prime the resulting catalog of biological tilings to serve as a multidisciplinary meeting point, offering a window for exploring selective pressures and trade-offs in anatomical and functional evolution, for interpreting historical examples of artificial tessellations, and for informing innovative approaches in design and architecture.

## Methods

### Defining biological tilings

To build a useful compendium of biological tessellations, we needed a functional definition suited to biological systems. In the biological literature, however, the functional roles of tessellated architectures have barely been considered (but see e.g. 16–18, 21, 25–28) and so no operable unifying definition exists. In mathematics, tessellations have a quite specific definition, referring to the covering of a Euclidean plane or the filling of 3D Euclidean space with elements (tiles or cells) of one or more geometric shapes, where the elements neither overlap nor have gaps between them (29). The problem with converting the mathematics definition to a biological one is that biogenic “tessellations” (i.e. of discrete abutting tissue elements) will always have some gap between individual elements, albeit perhaps microscopic. We therefore used the term *tiling* in our collection to distinguish these designs relative to mathematical tessellations where borders between elements are truly common (i.e. effectively fused).

Our team represents a diversity of disciplines involving biologists, computer scientists, engineers and designers, allowing us to examine biological tilings from a range of perspectives. We started by compiling a list of biological examples that loosely fit the mathematical definition, using scientific literature sources and search engines (e.g. Web of Knowledge, Scopus). Directly relevant search terms (e.g. “tessellation,” “tiling,” and “pattern”) yielded limited results from the literature, so we relied heavily on ad hoc and/or “popular science” approaches: solicited discussions with scientific experts, image searches (e.g. using Google Images, Google Lens, Pinterest) and explorations of diverse nonspecialist websites (e.g. Wikipedia, ×[formerly Twitter], nature photography blogs). Among these sources, the biological examples commonly considered to be “tessellations” varied hugely in their structure, ranging from bee honeycombs (i.e. hard walls enclosing spaces), to fish skins (i.e. hard scales overlapping like roof shingles), to skin and fur patternings (i.e. local variation in pigmentation, not structure).

The initial search characteristics resulted in a collection of more than 120 biological examples, which we used to determine a definition for biological tilings (and the variables used to characterize them). In particular, the nature of how tiles physically interact is fundamentally different between true mathematical tessellations and biological tilings. Whereas the former are, by definition, “edge-to-edge” tilings, where tiles share a margin, we found that biological tiles are often embedded in or isolated from each other by a softer linking or matrix material (a “joint” phase) (18, 22, 30–32). Unlike a true polygonal tessellation drawn on a page and the “inverse” tessellations we describe below, where adjacent cells have walls in common, tilings built from individual 3D objects can have tiles that abut but do not actually share an edge. As a result, the biological tilings we observed always exhibited some degree of overlap or gap (e.g. filled with body fluid or air), inherently disqualifying them as true/strictly mathematical tessellations. From our initial observations, we therefore established a base definition less strict than the mathematical one: biological tilings are structural motifs or patterns involving repeated arrangements of discrete “tiles” separated by a “joint” material.

Although many structural patterns are popularly referred to as tessellations, we feel our terminological distinction between tiling and tessellation is important for underlining that individual tiles are in fact separate entities, and therefore possibly capable of individual growth, movement or repair. The structural and mechanical properties implicit in our definition exclude a variety of natural structural patterns, commonly cited as tessellations. Honeycombs and other closed cell foams, for example, are effectively inverse tilings, with hard walls and a soft or missing interior (12, 14, 15). In our initial collection, we found that “inverse tessellations” were the most referenced examples of tessellations. These include spiderwebs; cellular tissue laminae (e.g. epithelia); porous shells (frustules) of diatoms; reticulated polypore fungi (e.g. *Hexagonia*); and the stomach lining of ruminants (33). Also excluded by our definition are patterns that are purely pigmentation-based, such as the variegated furs of giraffe or jaguar. In order to focus on tissue-level properties, we also excluded structures formed by groups of organisms, for example the spheroidal colonies of *Volvox* algae, colonial corals (e.g. *Hexagonaria percarinata*), and aggregate rafts of *Culex* mosquito eggs; these, however, could easily be included in future expansions of the collection (see Results and synthesis section). Lastly, we excluded broad patterns of general body segmentation (e.g. insect tagmata, vertebral columns, and skull bones of vertebrates), as their diversity across a huge number of organisms makes them challenging to classify universally.

Given the limited literature on biological tilings, we were often forced to infer aspects of tissue architecture solely from images and/or morphologies that seemed plausible based on closely related organisms. As a result, we focused primarily on categorical descriptions of tilings rather than quantitative ones, as discrete classifications could be most reliably applied across examples. In several cases, we discarded examples with limited and/or ambiguous anatomical data (e.g. silica tablets of discinid brachiopods, sclerotic plates of extinct worm-like *Microdictyon*). Many of our designations will surely benefit from reevaluation, as higher-resolution and 3D data are gathered to clarify whether examples are arrays of “tiles” or rather just tessellated patterns marked on surfaces (e.g. patterns on the surfaces of insect and brine shrimp eggs (34)).

### Classifying biological tilings

Starting from our more than 120 examples and removing cases that did not fit our refined definition, we arrived at 100 examples of biological tilings. Our examples represent varying numbers of species depending on available information, some referring to single species and others to a tiling type shared by members of a larger phylogenetic group. For example, “salak fruit epicarp” comprises the single species *Salacca zalacca*, whilst “ammonite shells” references the many thousand possible ammonite species (35, 36). We should note that our tessellation examples often may not represent a defining autapomorphy for an entire phylogenetic group, since there often is not enough information currently available to clarify this definitively. Instead, by using coarse and well-documented groupings rather than trying to tease apart intragroup variation from limited literature, we were better able to provide an overview of the variety of tilings in nature, while also highlighting less-explored tilings/taxa deserving more focused research in the future. The thought process behind our classification scheme is further discussed in the Synthesis section.

With the 100 examples of tilings, we organized them according to broader taxonomic groupings: plants, arthropods, annelids, mollusks, echinoderms, cartilaginous fishes, bony fishes, amphibians, sauropsids, and mammals. Additionally, we categorized according to an even broader taxonomic bin: plants, deuterostomes, protostomes, and other. To examine the variety and commonalities of the collected tilings, we devised a classification scheme, “coding” examples according to their material, morphological, and functional properties. In addition to our multidisciplinary working group, we involved experts for the clarification of the codings for specific examples where literature was limited or lacking. Our final codings, however, should be seen as works-in-progress and the tiling catalog as evolvable: new examples can be added and current examples refined as research becomes available (in both cases, hopefully sparked by the current work).

The following coding variables and their sub-variables were defined and modified over multiple iterations, in an effort to describe and encompass the diversity of collected tilings. A tiling example may be described by multiple sub-variables, as will be discussed in the following descriptions. For the remainder of the article, we refer the reader to the Supplementary material, which contains citations showing the tiled morphologies for each of our examples (Supplement Data S1).

#### Tile and joint materials

**Fig. 1.**
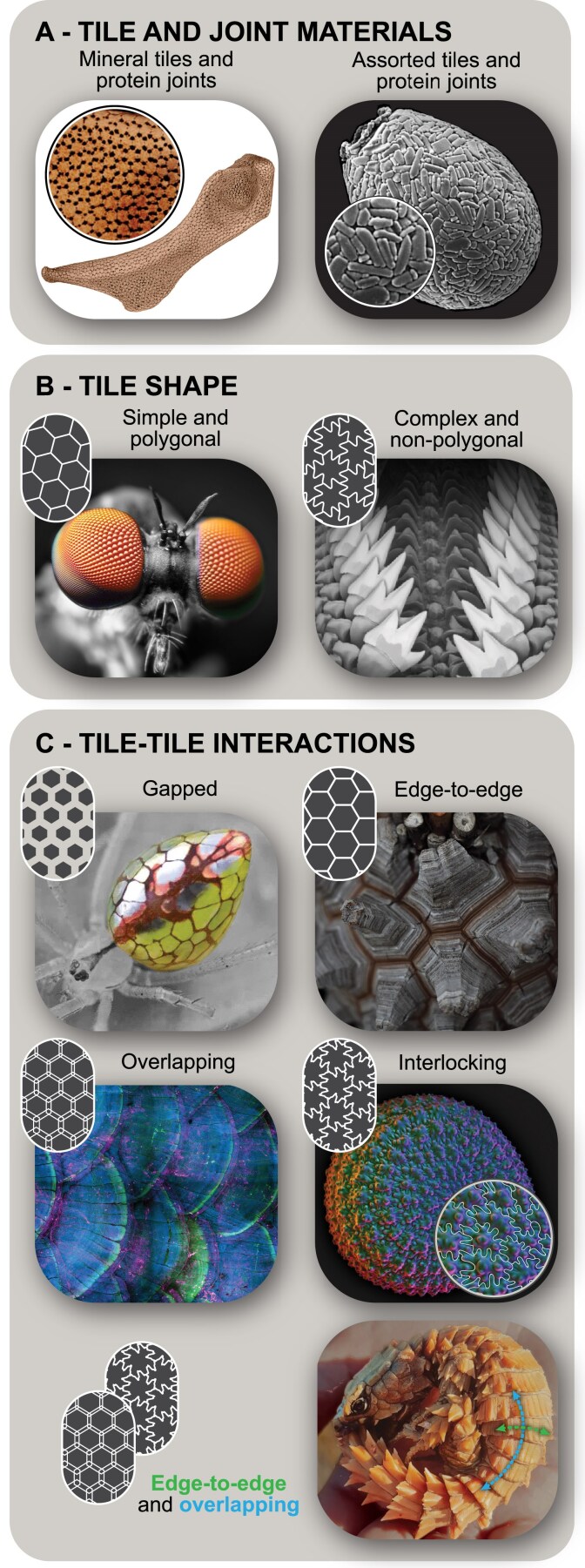
Tiling classification variables and sub-variables with examples, with simplified icons. A) Tile and joint materials: tile and joint constituents can involve mineral, protein, sugar or other materials, either homogeneously or in combination. For example, (left) the tessellated cartilage of sharks and rays comprises mineral/protein tiles and protein joints, whereas (right) agglutinate amoeba tests involve assorted tile materials and protein joints. B) Tile shape: (simple and polygonal) insect eye ommatidia; (complex and nonpolygonal) chiton radula teeth. Note, sub-variables for complexity (simple/complex) and geometry (polygonal/nonpolygonal) are nonexclusive. C) Tile-tile interactions: (gapped) mirror spider abdomen; (edge-to-edge) elephant's foot plant bark; (overlapping) fish scales; (interlocking) millet seed coat. Note, multiple types of interaction can exist in one specimen, for example (bottom right), the overlapping and edge-to-edge tiling of armadillo lizard scutes. All image usage rights can be found in Appendix at the end of the article.

The materials that make up the tiles and joints of a biological tiling play important roles in mediating tile–joint and tile–tile interactions, and therefore the functional roles the tiling can serve (Fig. 1A). Biological materials are typically composites of multiple components in various proportions (1, 2, 37), and therefore we code tile and joint materials only according to their dominant building blocks: (i) minerals (e.g. calcium phosphate, magnetite, silica, calcium carbonate); (ii) proteins (e.g. collagen, fibroin, sericin); (iii) sugars (e.g. chitin, cellulose, polysaccharides); and (iv) other materials (e.g. guanine, lignin, water). We code tile and joint materials separately, since these often have quite different material properties (e.g. stiffness, flexibility).

The tiles and joints may contain some similar components, even if their overall composition has key differences. For example, collagen is a common base component in both mineralized and unmineralized tissues in vertebrate animals (2, 38); in the tessellated cartilage of sharks and rays, both the tiles (tesserae) and unmineralized joints are collagenous, but the tesserae also contain mineral (calcium phosphate) (39). On the other hand, tile and joint materials can sometimes be entirely different in their composition. For example, testate amoebae, which cobble together or grow a shell-like test, can have “tiles” that comprise a variety of different minerals depending on availability, but they are all held together by an organic cement matrix (40). In many of our examples, there is limited published information about the exact material composition of the biological tilings, especially the joints. We therefore occasionally base codings on knowledge of related organisms. Since joint material and structure are typically more poorly described in the literature, the remaining variables focus more on the tiles themselves.

#### Tile shape

The mechanical behavior of tilings is determined in part by how tiles interact physically with each other, which is a function of tile shape (18, 30, 41) (Fig. 1B). Unfortunately, the features not visible externally are yet to be described for many biological tilings (e.g. overlaps, protrusions, or complex interlocks of tiles in the z-direction). For this reason, we focus on characterizing the shape of tiles according to their outlines when looking down on the tiled surface (i.e. a tile's projected shape on the XY plane); with increased future focus on biological tilings, ideally the 3D shape of tiles would also be considered as a distinguishing factor.

With regard to the complexity of the tile outline, we define (i) simple tiles as possessing only “small” internal angles (<180° vertex angles), whereas (ii) complex tiles possess at least one “large” internal angle (a vertex angle >180°), as occurs when a tile has a protrusion or angular process jutting off of one side. With regard to tile geometry, (iii) polygonal tiles have pointed corners; whereas (iv) nonpolygonal tiles have undulating or rounded edges. Tile complexity and geometry are not exclusive: a simple or complex tile can be either polygonal or nonpolygonal. For example, the compound eyes of insects involve simple, polygonal tiles (lenses packed in a regular hexagonal pattern), whilst the tongue-like radula of mollusks bears rows of complex, nonpolygonal teeth.

#### Tile–tile interactions

In addition to tile shape, the properties and function of a tiling are related to the nature of the interaction of its adjacent tiles, through either direct tile–tile contact or indirect tile–joint–tile contact (18, 41–43) (Fig. 1C). We define four types of interactions, in order of increasing tile–tile involvement. (i) Gapped interactions involve a visible gap between tiles, filled with the surrounding medium (air or water) or tissue (e.g. the intestines of the mirror spider can be squeezed between the tiled guanine plates of their abdomen). (ii) Edge-to-edge interactions include the abutment of simple tiles, but no interlocking or overlap, as seen in the corky plates of the elephant's foot plant stem. (iii) Overlapping interactions encompass tiles protruding from the surface, partially overlying each other, common for skins covered with plate-like scales, as in fishes and snakes. (iv) Interlocking interactions consist of abutting complex tiles sharing borders, with the edge shape of one tile forming the “negative” of the other, as seen in the jigsaw-puzzle-surfaces of millet seed coats. We consider “nesting” tilings to also be a form of interlocking, where each tile stacks completely into the next, as in deep-sea tubeworms (*Lamellibrachia*), where the cylindrical tube segments slot together like stacked cups.

Different types of tile–tile interactions sometimes exist in a single organism. For example, armadillo lizard scutes comprise belt-like transverse edge-to-edge bands, which form overlapping interactions longitudinally along the body. In 3D tilings, the nature of tile–tile interaction can differ between the top and bottom of tiles. For instance, tiles embedded in a membrane might be gapped on one side but edge-to-edge or overlapping on the other, as with tiles that jut up like islands (e.g. mammal and reptile osteoderms, elephant skin) or like plates anchored at one edge (e.g. fish and reptile scales).

#### Scale

**Fig. 2.**
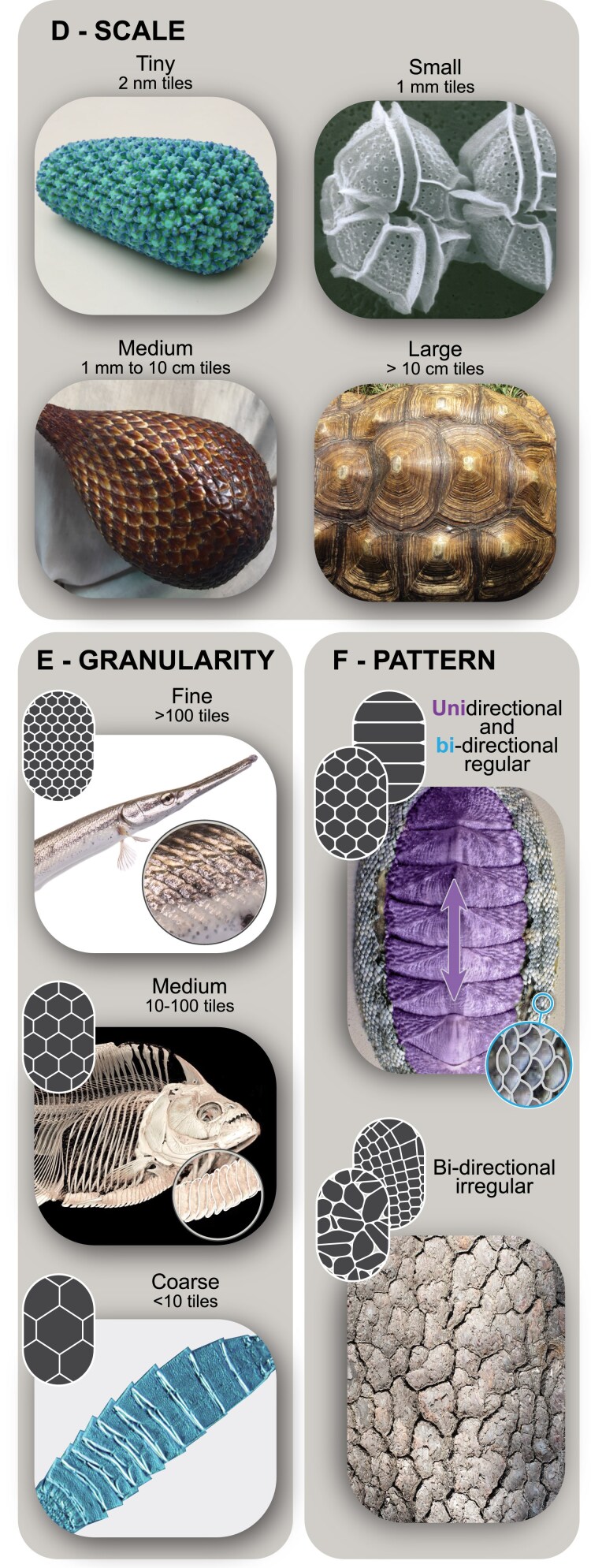
Tiling classification variables and sub-variables with examples, with simplified icons (cont.). D) Scale: (tiny) virus capsid; (small) dinoflagellate thecae; (medium) salak fruit epicarp; (large) turtle shell. E) Granularity: (fine) fish scales; (medium) keel scutes in piranhas; (coarse) mud dragon armor. F) Pattern: Note, multiple types of interaction can exist in one specimen, for example (unidirectional, regular) chiton shell plate and (bidirectional, regular) chiton girdle, (bidirectional, irregular) tree bark. All image usage rights can be found in Appendix at the end of the article.

Among biological tilings, the absolute size of individual tiles varies considerably, from nanometers to tens of centimeters (Fig. 2D). Detailed quantitative information on the scale and size variation of tiles in most biological tilings, however, does not exist. We therefore often determine tile size range based on the projected diameter of tiles from available images. From these, minimum tile size is estimated broadly, according to orders of magnitude: (i) tiny tiles are smaller than 10^1^ µm, as seen in virus capsids (the smallest in our collection, ∼2 nm per tile); (ii) small tiles fall between 10^1^ and 10^3^ µm (1 mm) (e.g. the thecae of dinoflagellates); (iii) medium tiles between 10^3^ and 10^5^ µm (1 mm to 10 cm), like the epicarp scales of the salak fruit; and (iv) large tiles being those larger than 10^5^ µm (10 cm), such as the carapace scutes of giant turtle shells, which are 10s of centimeters in diameter. Tilings where tile size visibly changes over the tiled area are coded as (v) tapering tilings.

#### Granularity

Regardless of absolute tile size, how tiles pack into an available space is a decisive factor in the architecture of a tiled surface (33, 41) (Fig. 2E). In a manner similar to sandpaper, we define granularity as the number of tiles relative to the area tiled, to account for some tilings covering entire organisms and others only portions of them. We estimated tiling granularity from available images, but these were often rare, low quality, and/or showed only the surfaces of tilings. As a result and to account for variability, granularity was described in three broad bins: (i) coarse-grain tilings comprise fewer than 10 tiles over the tiled area; (ii) medium-grain tilings comprise between 10 and 100 tiles; and (iv) fine-grain tilings comprise more than 100 tiles. Scales of various types can ensheath an organism's entire body, as in the coarse-grain armors of the tiny, invertebrate mud dragons (kinorhynchs) or the fine-grain ganoid-scale armor of gar fish. In contrast, other fish tilings only form single strips running down the body, as in the medium-grain keel scutes of serrasalmid fishes like piranha.

#### Pattern

The pattern of a tiling—its layout and regularity—also dictates how its tiles cover surfaces (Fig. 2F). With regard to a tiling’s layout, (i) unidirectional tilings are arranged along just one axis (i.e. a strip); whereas (ii) bidirectional tilings are matrices arranged along two axes. With regard to regularity, (iii) regular tilings are symmetrical and therefore predictable patterns, based on a regular grid; whereas (iv) irregular tilings are without symmetry, formal arrangement or periodicity, typically involving irregular tile shapes (see above). In extreme cases, this might involve just one tile shape, like the “einstein” tile discovered recently (44). As with the sub-variables for tile shape, those describing layout and regularity are not exclusive: both uni- and bidirectional tilings can be either regular or irregular. Also, even within a single organism, the tiling pattern can vary in different regions. For example, in chiton (armored mollusks) the large dorsal shell plates form a unidirectional and regular (coarse-grained) pattern, while the miniscule scales of the flexible, encircling girdle form a bidirectional (but also regular, fine-grained) pattern. In contrast to the regular arrays of plates and scales in chiton armor, tree bark exhibits irregular patterning.

#### Function

Taken together, the six previous variables generate a holistic description of the material and form of biological tilings. To close the loop and correlate observed structural and material factors with a structure's role (or roles) in an organism's ecology, we define a series of functional variables to capture a range of reported or inferrable functions. Defining the function of a tiling requires understanding how an organism uses it in nature, but for many of our examples, such information is limited. Our codes were therefore often informed hypotheses, based on available literature, observation and deduction: (i) structural support tilings strengthen the body and provide attachment for muscles and tendons (e.g. Fig. 1A); (ii) shielding tilings protect the body from external forces, as with armors (e.g. Fig. 1C); (iii) surface-regulating tilings create adhesion or friction (e.g. to grip or grasp) (e.g. Fig. 1B, right); (iv) sensing tilings function in light or infrared reception (e.g. Fig. 1B, left); (v) separation tilings partition external and internal environments, regulating flux of, for example, water, gas or heat (e.g. Fig. 2F, bottom); (vi) mobility tilings, through the collective motion of individual tiles, generate larger deformations and movement of the overall tessellated area, as in armored animals that roll their bodies (e.g. Fig. 1C, bottom right); (vii) optical tilings control the appearance of the structure or organism by reflecting or refracting light (e.g. Fig. 1C, top left). Again, these are not exclusive sub-variables: we found most biological tilings are used diversely, and so we often ended up coding them with more than one function (e.g. the mobility-shielding-separation tessellations of armadillo lizards and chiton; Figs. 1C, bottom right and 2F, top). These multipurpose functions are keys to the understanding of tilings and are further examined in the Synthesis section of this article.

### Comparing biological tilings

We used various statistical tools in R version 4.2.0 (45) to explore overall as well as taxon-specific patterns in the tiling variables. We used the multiple correspondence analysis (MCA) function from the “FactoMineR” package (46), which is designed to handle multivariate datasets of categorical variables (like ours). This technique, in a way similar to principal component analysis (PCA), looks for the dimensions with the largest variance in datasets. Whilst PCA depends directly on numerical data, MCA depends on categorical data that is transformed into pseudocontinuous data based on the relative frequencies of the categories (47).

The MCA function converted our qualitative data into a pseudocontinuous format using the following procedure. First, each variable (e.g. tile material, tile shape, pattern, function, etc.) was split into the number of sub-variables it contained (e.g. for tile material (tm), tm_mineral, tm_sugar, tm_protein, tm_other), and then sub-variable occurrences were categorized as present or absent for each example. For instance, an example with mineral tiles was coded present for “tm_mineral” and absent for “tm_sugar,” “tm_protein,” and “tm_other.” The MCA function in R split each binary sub-variable (e.g. “tm_mineral”) into two variables (e.g. “tm_mineral present” and “tm_mineral absent”) with 1s and 0s indicating presence and absence. Note that this makes sense for variables with more than two categories but it is redundant for binary variables, since the two new variables are the inverse of one another. Therefore, for clarity's sake, we ultimately only visualized the former variable (e.g. “mineral present”) in the graphs (see below). The new binary variables were used to calculate the relative frequency of occurrences ((total number of 1s)/(total number of examples)). Each “1” was then divided by the relative frequency of that variable (see Supplement Table S1 for a break-down of this process with *tile material - mineral*).

This transformed the binary dataset into a pseudocontinuous one, the values being lower the more occurrences of that category had been observed and higher when the occurrences of a category were rarer. The method of conversion of the dataset ultimately meant that after plotting the MCA's principal dimensions, biological examples which are characterized by rarer sub-variables were localized further from the origin. In this way, the graphs resulting from MCA can be useful for examining co-occurrence of sub-variables and the similarities between biological examples which share rare traits in a dataset, based on the clustering of points in MCA space. In our case, however, the high variance in our dataset (i.e. the small amount of variance explained by each principal dimension) meant that our MCA is most effective for examining outliers (i.e. less commonly occurring sub-variables with low variance; see the Results and synthesis section below).

While MCA highlights outliers and variability in the dataset, chord diagrams can be used to visualize specific links among variables, the frequency of their co-occurrence, and the commonality of a motif across biological examples. In both cases, measuring co-occurrences is important because it allows us to identify patterns of association between variables from across the spectrum of descriptors, providing insight into potential relationships that may not be immediately evident when analyzing variables in isolation or on a group-by-group basis. Chord diagrams show the pairwise relationships, whereas MCA summarizes the relationships among all variables. Using the same binary indicator variables as MCA, we created two-way frequency tables (1 and 0 × 1 and 0) and calculated the percentage of co-occurrences (1 and 1) for every pair of indicator variables. Our correlation analyses did not account for the nonindependence of data points due to phylogenetic relatedness, because of the way we defined our biological examples (see above) and/or because a resolved phylogeny was lacking for some groups in our data. As a result, we used chord diagrams to explore variable interactions without utilizing inferential statistics. We generated the chord diagrams using the “circlize” package (48), providing visual representations of the strength of co-occurrence among diverse sub-variables.

## Results and synthesis

**Fig. 3.**
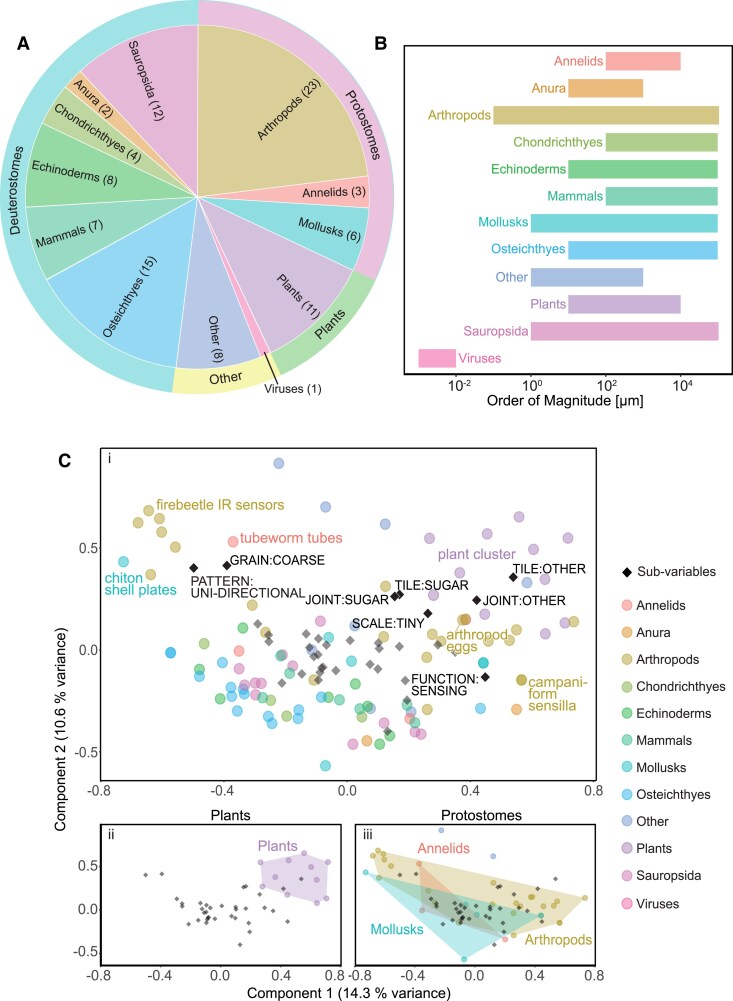
Example analyses of coded tiling data. A) Pie chart showing the number of examples in each of the four major taxonomic groups and their sub-groups. B) Bar chart showing the scale range of the tiled examples across taxonomic sub-groups. C) MCA of (i) all variables and examples, (ii) plant examples, and (iii) protostome examples. Sub-variables (black diamonds) and examples (colored dots) that suggest a trend or pattern have been labeled. Clustering of points can indicate a degree of relatedness of common factors e.g. among sub-variables and examples. The convex hulls highlight the broad variation across the arthropods and relatively small variation of the plant examples (see text for further explanation).

Our collection and formalized classification is a first glimpse into the broad variety of biological tilings, showing them to have evolved multiple times and in different forms across the Tree of Life; in diverse marine and terrestrial environments; and performing a host of different functions. In this way, our 100 collected examples show the tessellation motif to be a surprisingly inclusive collection of groups, including viruses (*n* = 1), plants (*n* = 11), protostomes (*n* = 34), and deuterostomes (*n* = 48) (Fig. 3A). These are surely not all the tilings that exist in nature: our collection is shaped by the availability of published work and particularly which systems have enjoyed focused attention from anatomists, structural biologists, microscopists, and wildlife photographers. For example, there is considerable and detailed morphological work on fish scales and armors (16, 17, 19–21, 49, 50). Similarly, we are more likely to be aware of examples from taxonomic groups where tilings are a shared, defining feature (e.g. squamate reptiles), and/or groups that have an especially large number of species (e.g. insects, fishes). Nearly a quarter of our tiling examples for instance, are from arthropods, their examples including diverse structures (e.g. egg cases, wings, and armors), diverse aquatic and terrestrial habitats, and diverse functions from shielding to optics (37, 51). Availability of data, of course, also guided what we accepted as “examples”: if distinct anatomies or detailed classifications/subtypes had not yet been described for a particular tiling type, we relied on coarser categorizations. For example, we drill down to several distinct reptile species-level tilings, but still include the general example “reptile osteoderms” (52). In these ways, our collection is certainly skewed by what the scientific community and our group of authors, who are most familiar with vertebrates and arthropods, know well. We therefore consider many of our examples as placeholders, inviting future, deeper investigation into variation. In the meantime, our collection can be a useful jumping off point for exploring how form and function vary and interrelate in biological tilings. In the paragraphs below, we sample several comparative data visualizations to demonstrate how underlying intra- and intergroup trends can be distilled from our tilings collection and can be explored on the accompanying website. Such pursuits begin to pave the way to identifying deeper correlative and causative links among the variables that characterize biological tilings and thereby framing core form–function relationships which can be used by other disciplines e.g. in designing and constructing artificial tessellations.

Exploring the range and variation of even a single variable can bring to light potential relationships among phylogeny, material, and/or function. The scale of individual tiles (Figs. 3B and Supplement Data S1), for example, varies widely across our collection, from nanometers (10^−3^ µm) to 10s of centimeters (10^5^ µm). It is interesting to not only consider tile size scale in the context of phylogeny and the number of organisms represented by a group but also organism size. Our single example of virus tiling, for example, represents the smallest tile size in our dataset (nanometer scale components) and given our dataset only having a single virus example, logically exhibits a narrow range of tile sizes. In contrast, arthropods represent the taxonomic group with the most examples of tilings in our collection, and also the largest tile scale range (10^−1^ to 10^5^ µm) (37). In these ways, the differences in the range of observed sizes between virus and arthropod structures surely relate to the number of examples we found for each group and size range of organisms, but potentially also to the architectures and functions of their tilings. Additionally, there is a clear overlap in the 10^2^ µm to 10^4^ µm size range despite the differences between the average organism size of the examples found in each taxonomic group. To demonstrate, among the organisms we sampled, most of the echinoderms are twice the size of the arthropods, and similarly mammals twice the size of echinoderms but the tiles’ size scale still overlap across the 10^2^ µm to 10^5^ µm range. Although all tiles in our collection have an upper limit of 10^5^ µm the lower limits differ widely among the groups. This group-specific variation we see already suggests constraints at play worthy of investigation. Is the common upper size for tiles in arthropods, echinoderms and mammals related to a limit on tile weight or on shape? For example, are larger tiles less effective for building complex surfaces? What is the relationship between tile size scale and organism body size? Are the differing lower limits for the three groups’ tiles a function of their different building materials or their phylogenetic history or both? The suggested limits can also point to gaps in our knowledge: no other example in our collection matches the very small scale tiling of the virus, yet their nanostructured capsid coat is particularly well-studied (53, 54). With the advent of nanotomography tools (55–57), future work may find more tiled structures at this architectural scale.

Beyond single variable explorations, multivariate approaches like MCA can help bring underlying patterns and structure in the dataset into relief (Fig. 3C and Supplement Figure S1). In MCA, points closer to the origin are more frequently observed and the clustering of variables or examples indicates common co-occurrence. The grouping of points, therefore, can potentially unveil characteristics that are diagnostic for a cluster or taxonomic group (although judging the similarities between sub-variables or biological examples solely by distance between points in MCA space should be approached cautiously, since the first two dimensions of our MCA only represent approximately 25% of the total variance). Our plant examples, for instance, share the common joint and tile material characteristic of bearing the organic polymer lignin. Their distance from the origin indicates this is a shared characteristic otherwise rare among the other collected examples. The plant cluster's proximity to particular variables—*sugar-based joint materials* and *tiny-scale* tessellations—points to a prevalence of these variables in the group, although they are not necessarily differentiating characteristics of plants (Fig. 3Cii). The association of *unidirectional* and *coarse-grain* tilings with a diversity of arthropods, chiton shell plates and tubeworm tubes (top left of Fig. 3Ci and iii) shows this architecture appears multiple times in protostome tilings, but is comparatively rare in the collection. The *sensing* function is similarly uncommon, but found in close proximity to examples that share the coding, like the fire beetle infrared sensors and insect campaniform sensilla. These analyzes are equally adept at exposing the rarity of features within a group or a lack of defining characteristic: in Fig. 3Ciii, for instance, the broad spread of the arthropod group (*n* = 23) is likely due to the wide variation of characteristics within the taxon, whereas the distance of the mollusk examples from each other is more due to there being only three mollusk examples in our collection. The diverse explanations of our data clusters underline the importance of keeping an eye on biological context when interpreting multivariate data: a broad point distribution does not necessarily mean a total lack of commonality but could still indicate disparity within a group (i.e. just because they are related does not mean they are completely similar, although some key characteristics may be shared). Examples may appear to be associated with a variable in one graph presentation, but actually have little link to it (e.g. some of the examples near *sensing* in Fig. 3Ci). With so much phylogenetic and morphological diversity in our collection, it makes sense that only a small fraction of the variance in our 70 variables is represented by the first two dimensions of our MCA (∼25%); rather than over-extracting meaning from point distances, clusterings, or distributions, the analysis’ strength is rather in spotlighting *questions* about commonalities, that can become calls for focused expansion and scrutiny of examples.

**Fig. 4.**
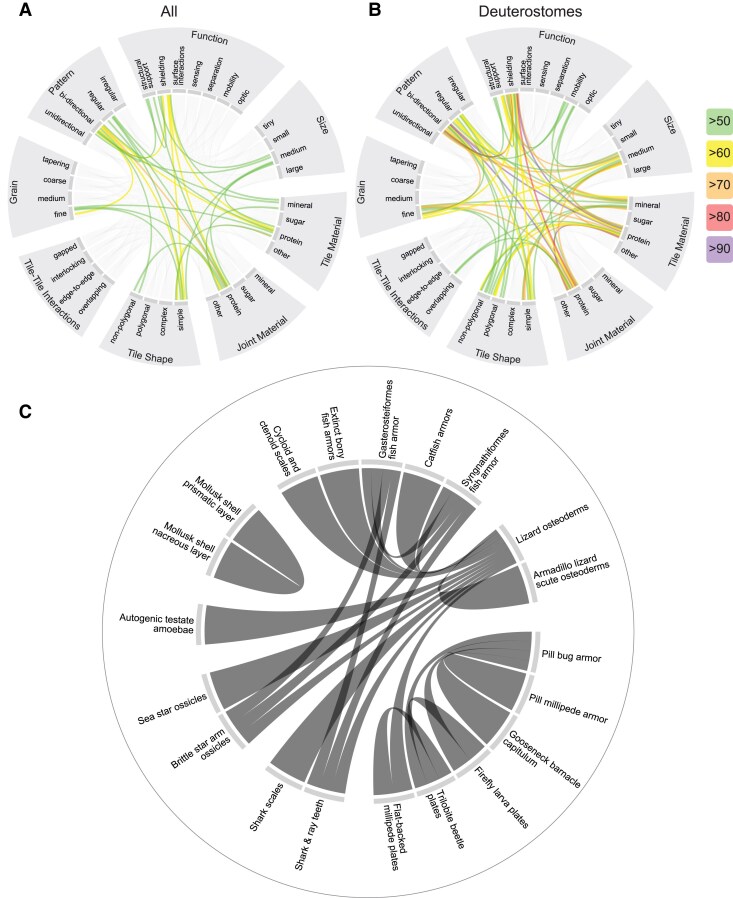
Chord diagrams, where linked elements indicate a co-occurrence, demonstrating the relationships between A) variables for all examples and B) variables for deuterostome examples. The colored lines represent the percentage of co-occurrence between 50 and 100% as indicated by the legend. In C), the relationships between examples with a correlation of 40% and above are shown. Note, chord width is not informative of the strength of correlation between variables nor examples.

We are particularly interested in the sub-variables that remain tethered together (that occur in a pairwise fashion), even across our diverse collected examples, as these may help us to identify the drivers that shape tiled structures during evolution (whether material, phylogenetic, or functional). Chord diagrams (Fig. 4A and B), for instance, in the particular patterning and coloring of their chords, can highlight specific links among variables, the frequency of their co-occurrence, and the commonality of a motif. Note in the chord diagram of our entire collection (Fig. 4A) that every variable (except *tile–tile interactions*) has colored lines emerging from at least one of its sub-variables, indicating the dominance of particular category co-occurrences. Some co-occurrences of sub-variables are exceptionally strong, occurring in more than 60% of cases (yellow and orange lines), despite the huge taxonomic variation. Such links start to frame cases of convergent evolution, for example, where a majority of our collected examples exhibit the triumvirate of *simple* tiles in *bidirectional* tilings used as *shielding*. The strong link between tile and joint materials that are protein-based illustrates how organic materials are regularly used to form the meshwork on which tilings are patterned and integrated (1, 2, 37, 58). Some co-occurrence patterns are dominant only within particular taxonomic groups: note the different latticework of the deuterostome chord diagram (Fig. 4B) relative to that of the entire dataset (Fig. 4A). For example, in contrast to the lack of stand-out tile–tile interactions in the complete dataset, *overlapping tiles* are a comparatively common motif among deuterostomes, although many sub-variables are emphasized in both graphs (e.g. medium-scale, fine-grain tilings). The emphasis of certain chords and heavy cross-correlation of sub-variables in the deuterostome graph is suggestive of a network of phylogenetic constraints favoring particular combinations of sub-variables. In this way, the deuterostome examples we collected are often *fine*-grained, *bidirectionally* arrayed, *regularly* patterned, *medium*-scale, *collagen*-based, with *simple* and *mineralized* tiles. In contrast, the constraints on tile polygonality and on function appear less stringent, since more character states exist. Fleshing out multivariate relationships like these—understanding how suites of sub-variables interact and map to functions—will be key to framing the selective pressures underlying the appearance (and recurrence) of distinct tiled patterns through evolution.

The overall similarity of particular tilings and the suites of characters that distinguish them can be explored by inverting the chord diagrams so that examples rather than variables populate the circumference (Fig. 4C). Again, connections between and among groups could suggest overall structure or structure–function convergence. The arthropod examples here show strong self-similarity (note the high degree of intragroup connectivity in the graph), a function of their shared joint/tile materials, but especially their particular tiling motif, where *simple*, *overlapping*, *nonpolygonal* tiles function in *structural support*, *shielding*, and *mobility*. Less intuitively, the links among the overlapping structures of bony fish dermal armor, brittle star armor, and shark/ray teeth speak to an overall structural similarity, even though these are disparate phylogenetic groups (bony fish, cartilaginous fish, and echinoderms) and the ecological functions of their tilings are quite different. In contrast, lizard osteoderms illustrate a “many-to-one” connection type, where chords extend outward like tree branches to link to diverse nonlizard examples, suggesting a broad convergence on osteoderm-similar design (note, however, that each connection represents a different cluster of coded variables). This also helps to highlight classifications in need of deeper exploration: do the many examples with similarity to lizard osteoderms indicate unappreciated osteoderm diversity? The many taxonomic groups linking to lizard osteoderms may in fact offer a roadmap for defining new morphofunctional classes of lizard armors.

**Fig. 5.**
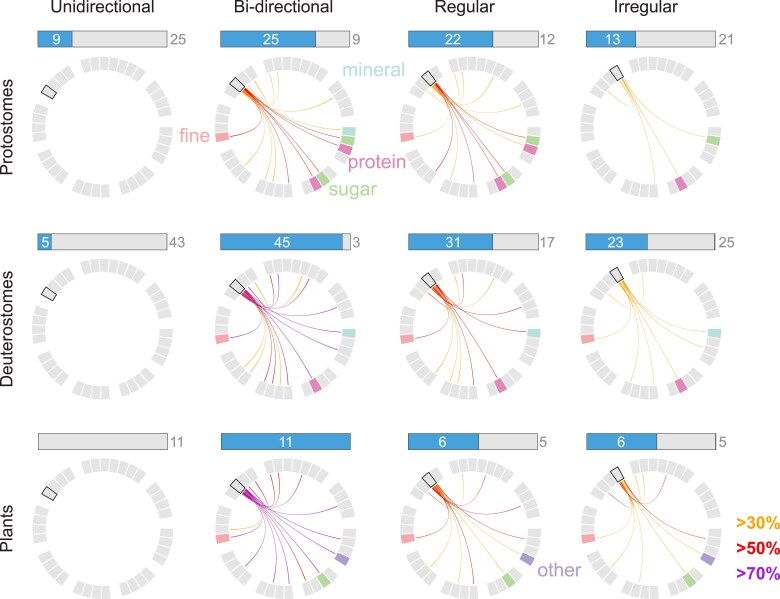
Linking prevalence and co-occurrence data for the four sub-variables relating to tiling pattern (unidirectional, bidirectional, regular, irregular) for protostomes, deuterostomes and plants (top, middle, and bottom rows, respectively). For each cell in the figure (e.g. Unidirectional-Protostome), the horizontal bar chart represents the proportion of presence (number in the box) and absence (number to the right of the box), while the chord diagram shows co-occurrence links among sub-variables (sub-variables position in chord diagram layout is as in Fig. 4A and B). The lines within the chord diagrams represent the percentage of co-occurrence, as indicated by the legend (30–49% orange, 50–69% red, >70% purple). In figure cells where examples were present (e.g. Unidirectional Protostomes), empty chord diagrams indicate little (<30%) or no category co-occurrence, in contrast to Unidirectional Plants where no examples were coded. Tile material and grain sub-variables have been highlighted to show co-occurrence motifs across groups; for example, note that some associations have heavy representation across all groups.

The correlations we observe in our analysis may mean different things to different taxonomic groups, especially since they are represented by different numbers of examples. A combination of data visualizations may be required: focusing on the *pattern* variable, for example, taking into account both the occurrence (pie charts) and dominance (line presence/color) of sub-variables, we can clarify whether a lack of lines in a chord diagram indicates rarity or indeed absence of a particular correlative relationship in a group, allowing for a more accurate assessment of cross-group variation. (Fig. 5). In these combined visualizations, *bidirectional* is clearly far more common than *unidirectional* in all three groups shown, just as *regular* tilings are more common than *irregular* tilings (albeit, less starkly so). Additionally, from the pie charts, an important observation can be made that *all* of our collected plant tilings are *bidirectional* and so the lack of *unidirectional* lines holds a different meaning in comparison to their absence in the other groups (i.e. where they are present in a few cases). Furthermore, although *regular* and *irregular* tilings are equally prevalent in some cases (plants) and the chord branching patterns seem to be roughly similar across all groups, the line colors are a lot less intense in some cases, particularly in the *irregular* protostome and deuterostome examples. Focusing more specifically on the constellation of sub-variables linked by chords can even lead to the identification of canonical sub-variables for individual groups and therefore paths of trait evolution. For instance by looking at the material segment of the chord diagrams, groups-specific motifs become obvious: protostomes have strong correlations with *sugar* and *protein*, deuterostomes with *mineral* and *protein*, and plants with *sugar* and *other*. In contrast, some associations have heavy representation across all groups, as evidenced by the many chords linking to *fine-grain* tilings.

## Conclusion/outlook

In summary, our analysis of biological tilings has demonstrated the taxonomic breadth and defined characteristics of this diverse motif, as well as highlighting less appreciated examples that can provide more nuance to this area of research. By creating a flexible and functional morphospace, we can add to the collection and refine previously existing examples with the goals of further clarification, development, and exploration. Our catalog can easily be expanded in the future to include additional examples, including tessellations formed by groups of organisms (see Methods) or even architectural examples (e.g. 11, 59, 60) and synthetic chemical tessellations (61, 62). Similarly, the catalog's framework delimits sub-variables that can drive targeted future exploration, to fill gaps we noted in current knowledge. For example, data on the exact material composition, quantitative descriptors of form (subsurface morphologies, size, and scale) and functionality of biological tilings are sorely needed for less studied taxonomic groups. The multivariate morphofunctional space we have built for biological tilings also allows us to examine similarities and differences among groups in a broader context and to raise more fundamental questions: why do some sub-variables dominate over others in certain taxonomic groups? Why are certain variable combinations more favored (e.g. bidirectional and regular tilings) and in what contexts do they appear? How could phylogeny be incorporated into future analysis to answer this? Which form-function relationships presented by biological tilings might lead to solutions for manufacturing design challenges, as with nacre-inspired fibers (63), chiton-inspired armors (25), and spider sensilla-inspired sensors (64)?

By framing and organizing the natural diversity of biological tilings, this catalog provides a new language for exploring the overarching formal principles defining biological tilings. Since biological tiled structures represent evolutionary solutions to particular problems (e.g. structural support), our collection offers tractable starting points (i.e. natural morphologies as “formal stereotypes” sensu (65)) for practical investigations of tessellation design potential. The features we have assembled as definitions for particular biological tilings can be translated into computational or generative design rules to guide fabrication of physical prototypes in design research (66–69). For example, by 3D printing solid tiles on prestressed textiles (70, 71), we can methodically examine the parameter space of hard and soft tissue interactions. Similarly, understanding the relationship between tessellations and surfaces can give us “alphabets” for providing new functional structures. These abstract representations of tessellations can then be altered parametrically to explore fine links between structure and performance, experimenting also with combinations and patterns that evolution has either weeded out or not yet arrived upon. In the parametric garment design project “*embrace3*” (72), tessellation is used as a strategy to design contextual and individualized surfaces for women who suffer from breast cancer and undergo a mastectomy. Tessellations allow the construction of asymmetrical surfaces which take into account a body topology which is currently not considered in the fashion industry.

**Fig. 6.**
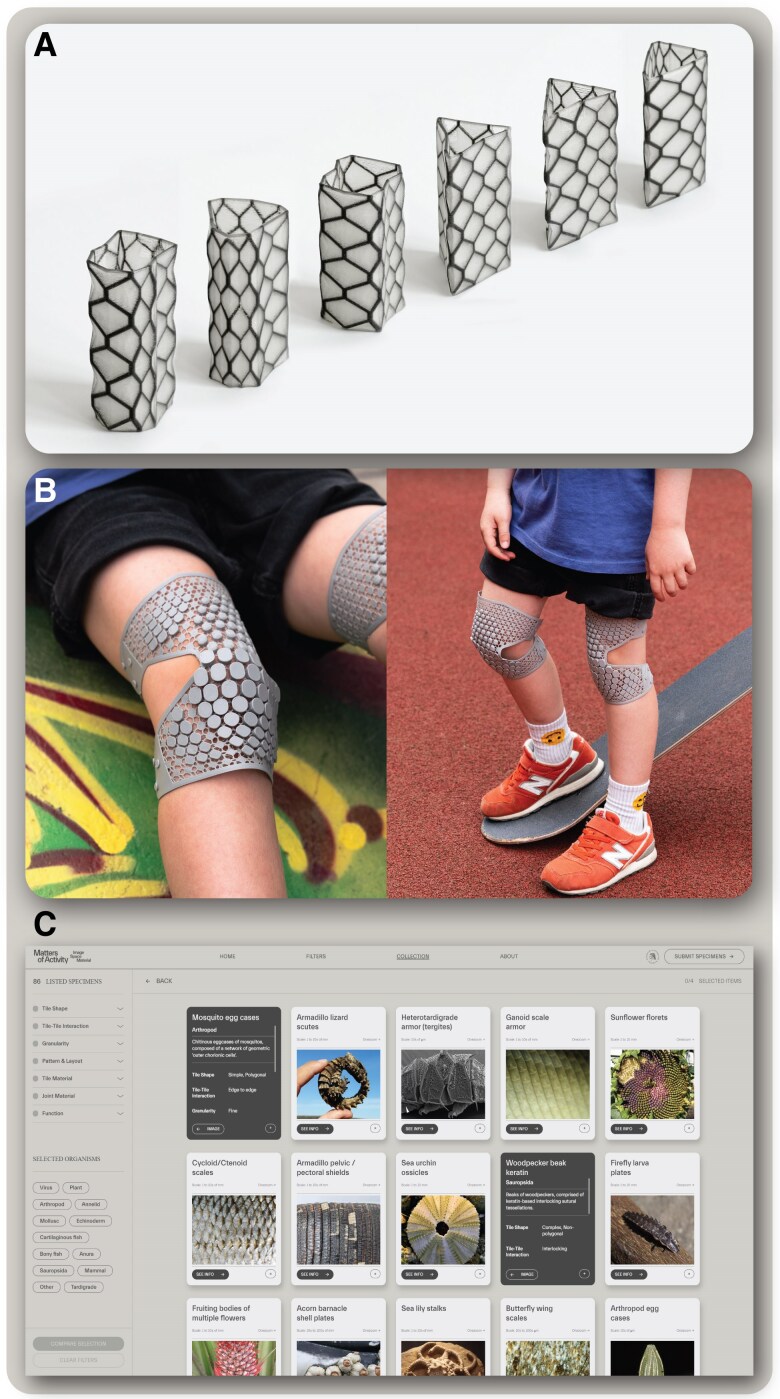
Application of the tessellation database. A) Diverse 3D-printed containers and B) knee pads, using the structural design of the boxfish carapace to inform tailorable methods for tiling complex surfaces (Designs: Felix Rasehorn, knee pad images: Leon Laskowski). C) Screenshot of the interactive TMS website where example cards display images and information that can be queried, filtered, and compared.

The architectural laboratory provided by tessellation systems also gives unique chances for design evolution through “performance” testing, where the material behavior computes the final shape or decides successful designs, rather than digital simulation (Material computation, sensu (73)) (Fig. 6A). The inherent activity and modularity of tessellations, for example, can be used to design individualized protective gear for children that is adaptable during growth and reactive to different needs of protection based on specific sports or ergonomic features (Fig. 6B). In this way, tessellation as a principle can encourage a culture of participation in design, for children to adapt their protection and even produce their own monomaterial gear in manufacturing spaces (e.g. MakerLabs) or at home. The abstract translation of features and generation of detailed physical prototypes (e.g. contrasting formal stereotypes with “evolved” designs) thereby allow comparison of diverse morphotypes and their performance. This provides clarity for interpreting the evolution of certain anatomies and anomalies in nature, where biology-informed design research can back-translate into actual product development with context-sensitive and adaptive design features of tessellated surfaces.

In these ways, our tiling catalog, coupled with additive manufacturing and generative design approaches offers an effective and interactive platform for transdisciplinary exchange and the evaluation of material behavior. To facilitate versatile conversations among disciplines, the companion website to this article (https://tessellated-materials.mpikg.mpg.de/; Fig. 6C) prioritizes the accessibility of tiling examples to a spectrum of backgrounds. Using a physical collection of childhood “Safari Cards” as inspiration, we present the catalog on the website as a series of digital information cards, showcasing illustrative images, links demonstrating phylogenetic placement (74) and relevant research papers. The information cards offer a visual and interactive platform for exploration, with the help of selection filters and a “click and compare” function where three examples can be compared side by side. We hope that the engaging, adaptable and content-rich design features of the website will encourage its use as a research tool and as an inspiration springboard for future projects with wider, multidisciplinary scope, asking vital questions beyond each individual field's traditional perspective.

## Supplementary Material

pgaf046_Supplementary_Data

## Data Availability

The complete tiling table—listing biological examples, their morphological codings, and relevant literature—is available as Dataset S1 with references provided in the Supplement Reference List for Data S1. This coding table is also available in an interactive visual format on the work's companion website: https://tessellated-materials.mpikg.mpg.de. The coded table and R script for data analyses are available to download at https://doi.org/10.17617/3.FYTTQ8.

## Appendix

**Appendix:** Figure image usage rights

**Table:**

Figure	Panel	Image Rights
1	All	Iconography, Felix Rasehorn (co-author)
1	A	Tesserae, adapted from Mason Dean (co-author)
1	A	*Netzelia oviformis*, adapted from Todorov M, Bankov N (2019) An atlas of sphagnum-dwelling testate amoebae in Bulgaria. Advanced Books. https://doi.org/10.3897/ab.e38685. Licensed under the Creative Commons Attribution License (CC BY 4.0) https://creativecommons.org/licenses/by/4.0/.
1	B	Compound eyes of a robber fly - (*Holcocephala fusca*), adapted from Thomas Shahan, background has been grayed out, https://www.flickr.com/photos/7539598@N04/3085177911/. © Thomas Shahan 2008, all rights reserved. Permission for re-use should be sought from the rights-holder.
1	B	Chiton radula (*Cryptochiton* sp.), adapted from James Weaver, http://mass.bio/m/G-Biomin.html. © James Weaver 2025 all rights reserved. Permission for re-use should be sought from the rights-holder.
1	C	Mirror spider, adapted from Nicky Bay, background has been grayed out, https://www.flickr.com/photos/nickadel/16432971400. © Nicky Bay 2015, all rights reserved. Permission for re-use should be sought from the rights-holder.
1	C	Elephant's Foot/*Dioscorea elephantipes*, adapted from Juuyoh Tanaka via Flickr. Licensed under the Creative Commons Attribution License (CC BY 2.0) https://creativecommons.org/licenses/by/2.0/.
1	C	Zebrafish Scales, adapted from Daniel Castranova, National Institute of Child Health and Human Development/NIH via Flickr, Licensed under the Creative Commons Attribution License (PDM 1.0) https://creativecommons.org/publicdomain/mark/1.0/deed.en.
1	C	Complex suture geometries in a plant seed coat, adapted from James Weaver, http://www.colorsem.com/P/C.html. © James Weaver, all rights reserved. Permission for re-use should be sought from the rights-holder.
1	C	Armadillo Girdled Lizard, adapted from Zoe Du Plessis, https://www.inaturalist.org/photos/328899523?size=original. Licensed under the Creative Commons Attribution License (CC BY 4.0) https://creativecommons.org/licenses/by/4.0/.
2	D	3D model of the HIV capsid, adapted from NIAID via Flickr. Licensed under the Creative Commons Attribution License (CC BY 2.0) https://creativecommons.org/licenses/by/2.0/.
2	D	Scanning electron microscope image of dinoflagelates, adapted from CSIRO via Wikimedia Commons. Licensed under the Creative Commons Attribution License (CC BY 3.0) https://creativecommons.org/licenses/by/3.0/deed.en.
2	D	Salak, adapted from Andre Hosper via inaturalist. Licensed under the Creative Commons Attribution License (CC BY 4.0) https://creativecommons.org/licenses/by/4.0/.
2	D	Turtle shell, adapted from dawsonlm via Flickr, Ben Townsend, Licensed under the Creative Commons Attribution License (CC BY 2.0), https://www.flickr.com/photos/bwtownsend/4887089996/
2	E	Juvenile Shortnose Gar (*Lepisosteus platostomus*), adapted from Sam Stukel via Flickr. Licensed under the Creative Commons Attribution License (PDM 1.0) https://creativecommons.org/publicdomain/mark/1.0/deed.en.
2	E	*Serrasalmus hastatus* UMMZ ventral, adapted from Matthew Kolmann and Ramon Nagesan. © Matthew Kolmann and Ramaon Nagesan 2022, all rights reserved. Permission for re-use should be sought from the rights-holder.
2	E	*Echinoderes hwiizaa*, adapted from Hiroshi Yamasaki & Shinta Fujimoto, Two new species in the *Echinoderes coulli* group (Echinoderidae, Cyclorhagida, Kinorhyncha) from the Ryukyu Islands, Japan. ZooKeys 382: 27–52. Background has been removed. Image provided via Wikimedia Commons as part of a collaboration between Wikispecies and Zookeys. Licensed under the Creative Commons Attribution License (CC BY 3.0) https://creativecommons.org/licenses/by/3.0/deed.en.
2	F	*Chiton tuberculata*, adapted from James St. John via Flickr, overlay and arrows have been added. Licensed under the Creative Commons Attribution License (CC BY 2.0) https://creativecommons.org/licenses/by/2.0/.
2	F	Pine bark, adapted from Creativity103 via Flickr. Licensed under the Creative Commons Attribution License (CC BY 2.0) https://creativecommons.org/licenses/by/2.0/.
6	A	Diverse 3D-printed containers, adapted from Felix Rasehorn (co-author)
6	B	Boxfish-informed knee patch design, adapted from Felix Rasehorn (co-author) and Leon Laskowski
6	C	Screenshot of TMS Website, Authors of this paper, https://tessellated-materials.mpikg.mpg.de
