# Supplementary material for: Tiled material systems: Exploring biodiversity and multifunctionality of a universal and structural motif
Source: PNAS Nexus. 2025 Nov 11;4(11):pgaf046. doi: 10.1093/pnasnexus/pgaf046 (PMC12604013; doi:10.1093/pnasnexus/pgaf046)
Supplement: pgaf046_Supplementary_Data [file pgaf046_supplementary_data.zip › Appendix.pdf]

SI Appendix

Contents

Table S1: Break down of MCA process with tm_mineral example 2
Figure S1: MCA with all plots labeled 4
Reference list for Data S1 5

Table S1: Break down of MCA process with tm_mineral example

	Tile material (variable)							
Alphanumeric	tm_mineral (binary sub-variable)	tm_mineral_present	tm_mineral_absent	tm_mineral_present (pseudo-continuous)	tm_mineral_absent (pseudo-continuous)	tm_sugar	tm_protein	tm_other
VI01	absent	0	1	0.00	2.50	absent	present	absent
PL01	absent	0	1	0.00	2.50	present	absent	present
PL02	absent	0	1	0.00	2.50	present	absent	present
PL03	absent	0	1	0.00	2.50	present	absent	present
PL04	absent	0	1	0.00	2.50	present	absent	present
PL05	absent	0	1	0.00	2.50	present	absent	present
PL06	present	1	0	1.67	0.00	present	absent	present
PL07	absent	0	1	0.00	2.50	present	absent	absent
PL08	absent	0	1	0.00	2.50	present	absent	absent
PL09	absent	0	1	0.00	2.50	present	absent	present
PL10	absent	0	1	0.00	2.50	present	absent	present
PL11	present	1	0	1.67	0.00	present	absent	absent
EU01	absent	0	1	0.00	2.50	present	absent	absent
EU03	present	1	0	1.67	0.00	present	absent	absent
EU04	present	1	0	1.67	0.00	present	present	absent
EU05	present	1	0	1.67	0.00	present	absent	absent
EU06	absent	0	1	0.00	2.50	present	absent	absent
EU07	present	1	0	1.67	0.00	absent	present	absent
EU08	present	1	0	1.67	0.00	absent	absent	absent
TA01	absent	0	1	0.00	2.50	present	absent	absent
AR01	present	1	0	1.67	0.00	present	present	absent
AR02	present	1	0	1.67	0.00	present	present	absent
AR03	present	1	0	1.67	0.00	present	present	absent
AR04	present	1	0	1.67	0.00	present	present	absent
AR05	present	1	0	1.67	0.00	present	present	absent
AR06	present	1	0	1.67	0.00	present	absent	present
AR07	absent	0	1	0.00	2.50	present	present	absent
AR08	absent	0	1	0.00	2.50	present	present	absent
AR09	absent	0	1	0.00	2.50	present	present	absent
AR11	absent	0	1	0.00	2.50	present	present	absent
AR12	absent	0	1	0.00	2.50	present	present	absent
AR13	absent	0	1	0.00	2.50	present	absent	absent
AR14	absent	0	1	0.00	2.50	present	present	absent
AR15	absent	0	1	0.00	2.50	present	present	absent
AR16	absent	0	1	0.00	2.50	present	present	present
AR17	absent	0	1	0.00	2.50	present	present	absent
AR18	absent	0	1	0.00	2.50	present	present	absent
AR19	absent	0	1	0.00	2.50	present	present	absent
AR20	absent	0	1	0.00	2.50	present	present	absent
AR21	present	1	0	1.67	0.00	present	present	absent
AR22	absent	0	1	0.00	2.50	present	present	absent
AR23	absent	0	1	0.00	2.50	present	present	absent
AR24	present	1	0	1.67	0.00	absent	present	absent
AN01	absent	0	1	0.00	2.50	absent	present	absent
AN02	present	1	0	1.67	0.00	absent	absent	absent
AN03	absent	0	1	0.00	2.50	present	present	absent
MO01	present	1	0	1.67	0.00	present	absent	absent
MO02	present	1	0	1.67	0.00	absent	absent	absent
MO03	present	1	0	1.67	0.00	absent	absent	absent
MO04	present	1	0	1.67	0.00	present	present	absent
MO05	present	1	0	1.67	0.00	present	present	absent
MO06	present	1	0	1.67	0.00	absent	absent	absent
EC01	present	1	0	1.67	0.00	absent	present	absent
EC02	present	1	0	1.67	0.00	absent	present	absent
EC03	present	1	0	1.67	0.00	absent	present	absent
EC04	present	1	0	1.67	0.00	absent	present	absent
EC05	present	1	0	1.67	0.00	absent	present	absent
EC06	present	1	0	1.67	0.00	absent	present	absent
EC07	present	1	0	1.67	0.00	absent	present	absent
EC08	present	1	0	1.67	0.00	absent	present	absent
CH01	present	1	0	1.67	0.00	absent	present	absent
CH02	present	1	0	1.67	0.00	absent	present	absent
CH03	present	1	0	1.67	0.00	absent	present	absent
CH04	present	1	0	1.67	0.00	absent	present	absent
OS01	present	1	0	1.67	0.00	absent	present	absent
OS02	present	1	0	1.67	0.00	absent	present	absent
OS03	present	1	0	1.67	0.00	absent	present	absent
OS04	present	1	0	1.67	0.00	absent	present	absent
OS05	present	1	0	1.67	0.00	absent	present	absent
OS06	present	1	0	1.67	0.00	absent	present	absent
OS07	present	1	0	1.67	0.00	absent	present	absent
OS08	present	1	0	1.67	0.00	absent	present	absent
OS09	present	1	0	1.67	0.00	absent	present	absent
OS10	present	1	0	1.67	0.00	absent	present	absent
OS11	present	1	0	1.67	0.00	absent	present	absent
OS12	present	1	0	1.67	0.00	absent	present	absent
OS13	absent	0	1	0.00	2.50	absent	present	absent
OS14	present	1	0	1.67	0.00	absent	present	absent
OS15	present	1	0	1.67	0.00	absent	present	absent
AM01	present	1	0	1.67	0.00	absent	present	absent

AM02	absent	0	1	0.00	2.50	absent	present	absent
SA01	absent	0	1	0.00	2.50	absent	present	absent
SA02	present	1	0	1.67	0.00	absent	present	absent
SA03	present	1	0	1.67	0.00	absent	present	absent
SA04	present	1	0	1.67	0.00	absent	present	absent
SA05	absent	0	1	0.00	2.50	absent	present	absent
SA06	present	1	0	1.67	0.00	absent	present	absent
SA07	absent	0	1	0.00	2.50	absent	present	absent
SA08	absent	0	1	0.00	2.50	absent	present	absent
SA09	present	1	0	1.67	0.00	absent	present	absent
SA10	absent	0	1	0.00	2.50	absent	present	absent
SA11	present	1	0	1.67	0.00	absent	present	absent
SA12	present	1	0	1.67	0.00	absent	present	absent
MA01	absent	0	1	0.00	2.50	absent	present	absent
MA02	absent	0	1	0.00	2.50	absent	present	absent
MA03	present	1	0	1.67	0.00	absent	present	absent
MA04	present	1	0	1.67	0.00	absent	present	absent
MA05	present	1	0	1.67	0.00	absent	present	absent
MA06	absent	0	1	0.00	2.50	absent	present	absent
MA07	present	1	0	1.67	0.00	absent	present	absent
p (relative frequency of presence)		0.6	0.4					

Figure S1: MCA with all plots labeled

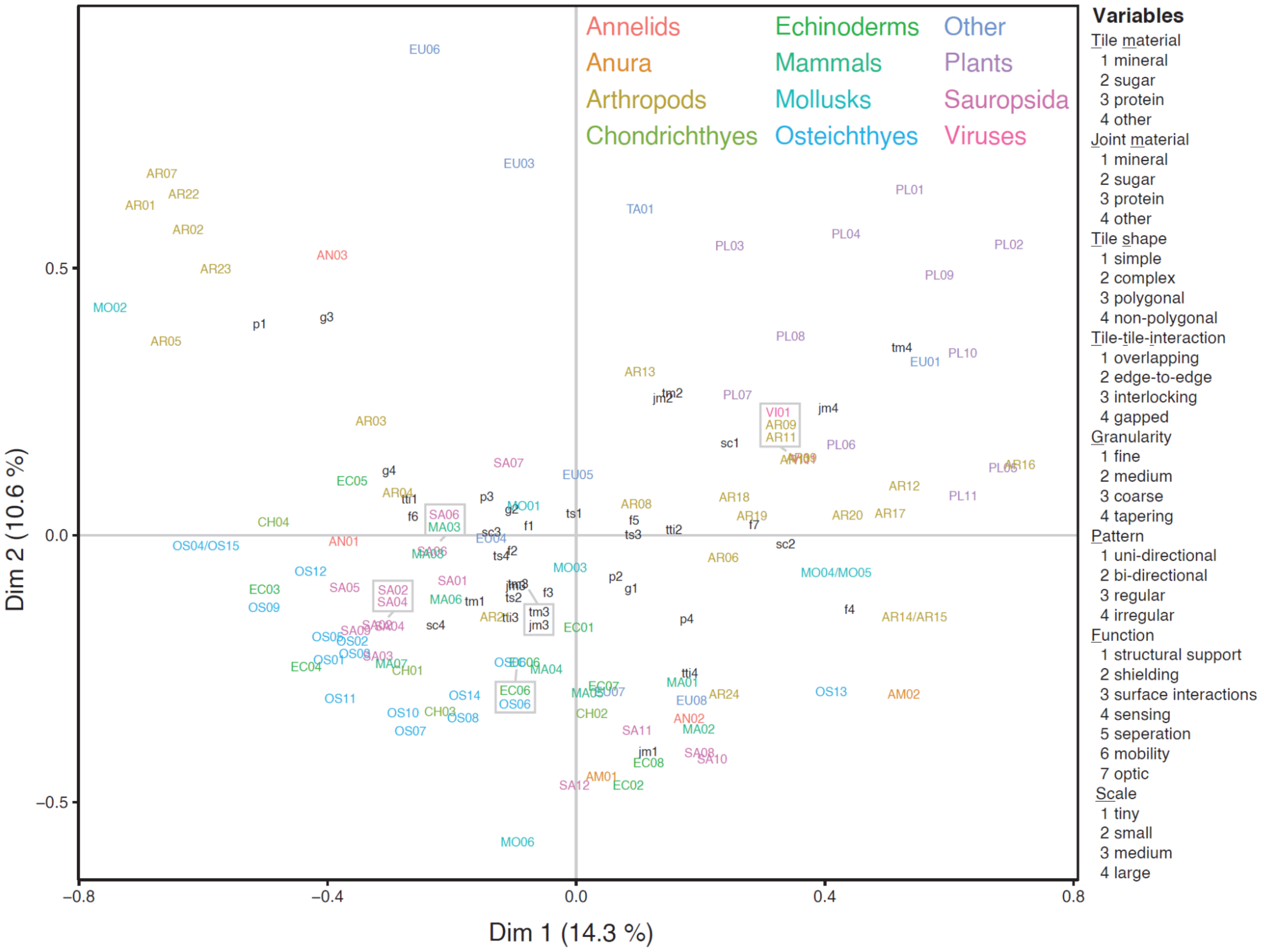

Reference list for Data S1

1. Mueller, J., 2018. Picornaviridae – Zum Gestalten von Modell- und Visualisierungskonzepten in der Virologie. Berlin: Humboldt-Universität zu Berlin, Exzellenzcluster Bild Wissen Gestaltung. ISBN 978-3-00-060929-9.
2. Twarock, R., Hendrix, R.W., 2006. Crosslinking in viral capsids via tiling theory. J. Theor. Biol. 240, 419–424. https://doi.org/10.1016/j.jtbi.2005.10.001
3. Krupovic, M., Koonin, E.V., 2017. Multiple origins of viral capsid proteins from cellular ancestors. Proc. Natl. Acad. Sci. USA 114 (12), E2401–E2410. https://doi.org/10.1073/pnas.1621061114
4. DigiMorph Staff, 2002. Ananas comosus (pineapple) [Specimen CT scan]. DigiMorph Project, University of Texas at Austin. Available at: http://digimorph.org/specimens/Ananas_comosus/
5. Okimoto, M.C., 1948. Anatomy and Histology of the Pineapple Inflorescence and Fruit. Bot. Gaz. 110 (2), 217–231. https://doi.org/10.1086/335530
6. Sparshott, E.N., 1935. Observations on the formation, development, and structure of the tuber of Testudinaria elephantipes, and on the origin of the vegetative shoot. J. Linn. Soc. Bot. 49 (332), 593–610. https://doi.org/10.1111/j.1095-8339.1935.tb01341.x
7. Dörken, V.M., Zhang, Z.X., Mundry, I.B., Stützel, T., 2011. Morphology and anatomy of male cones of Pseudotaxus chienii (W.C. Cheng) Taxaceae. Flora 206 (5), 444–450. https://doi.org/10.1016/j.flora.2010.08.006
8. Worsdell, R.H., 1954. The female reproductive organs of conifers and taxads. Biol. Rev. 29 (2). https://doi.org/10.1111/j.1469-185X.1954.tb01515.x
9. Lim, T.K. (2012). Salacca zalacca. In: Edible Medicinal and Non-Medicinal Plants. Springer, Dordrecht. https://doi.org/10.1007/978-90-481-8661-7_57
10. Supapvanich, S., Megia, R. & Ding, P., 2011. Salak (*Salacca zalacca* (Gaertner) Voss). In: *Postharvest Biology and Technology of Tropical and Subtropical Fruits: Mangosteen to White Sapote*. Woodhead Publishing, pp. 334–352. https://doi.org/10.1533/9780857092618.334
11. Antreich, S.J., Xiao, N., Huss, J.C., Horbelt, N., Eder, M., Weinkamer, R., Gierlinger, N., 2019. The puzzle of the walnut shell: A novel cell type with interlocked packing. Adv. Sci. (Weinh.) 6 (11), 1900644. https://doi.org/10.1002/advs.201900644
12. Hernandez, R., 1984. Distribution of Portulaca oleracea L. (Portulacaceae) subspecies in Florida. Systematic Botany, 9(2), pp. 175–181. Available at: https://www.jstor.org/stable/23909826
13. C. Gao, B. P. J. Hasseldine, L. Li, J. C. Weaver, Y. Li, Adv. Mater. 2018, 30, 1800579. https://doi.org/10.1002/adma.201800579
14. Hernandez, R., 1981. The seed surface morphology and cytology of six species of Portulaca (Portulacaceae). Systematic Botany, 6(4), pp. 349–358. Available at: https://www.jstor.org/stable/4033785
15. Bally, P. R. O., Horwood, F. K., & Lavranos, J. J., 1975. A Monograph of the Genera Pseudolithos & Whitesloanea. The National Cactus and Succulent Journal, 30(4), 88–93. http://www.jstor.org/stable/42792004

16. Windle, W.S., 1889. Fibres and raphides in fruit of Monstera. Botanical Gazette, 14(3), pp. 67–69. https://doi.org/10.1086/326385
17. Seiler, G.J. (1997). Anatomy and Morphology of Sunflower. In Sunflower Technology and Production, A.A. Schneiter (Ed.). https://doi.org/10.2134/agronmonogr35.c3
18. Junikka, L. (1994). Survey of English Macroscopic Bark Terminology. IAWA Journal, 15(1), 3-45. https://doi.org/10.1163/22941932-90001338
19. Pearce, P., 1978. Structure in Nature is a Strategy for Design. Cambridge, MA: MIT Press.
20. Hansen, G. H. and Flaim, G. (2007). Dinoflagellates of the Trentino province, Italy. Journal of Limnology, 66(2), 107. https://doi.org/10.4081/jlimnol.2007.107
21. Martone, P.T., Estevez, J.M., Lu, F., Ruel, K., Denny, M.W., Somerville, C. and Ralph, J., 2009. Discovery of lignin in seaweed reveals convergent evolution of cell-wall architecture. Current Biology, 19(2), pp.169–175. https://doi.org/10.1016/j.cub.2008.12.031
22. Denny, M.W. & King, F.A., 2016. The extraordinary joint material of an articulated coralline alga. I. Mechanical characterization of a key adaptation. Journal of Experimental Biology, 219(12), pp.1833–1842. https://doi.org/10.1242/jeb.138859
23. DiBartolo-Cordovano, R., Marks, C., Sprick, A. and Kingsley, R., 2009. Calcium Carbonate Test Formation in the Ciliate Coleps. Microscopy and Microanalysis, 15(S2), pp.910–911. Available at: https://doi.org/10.1017/S1431927609094963
24. Elsevier, 2012. Figure 1 from 'S104784771200319X'. [online] Available at: https://ars.els-cdn.com/content/image/1-s2.0-S104784771200319X-gr1.jpg
25. Triantaphyllou, M.V., Baumann, K.-H., Karatsolis, B.-T., Dimiza, M.D., Psarra, S., Skampa, E., Patoucheas, P., Vollmar, N.M., Koukousioura, O., Katsigera, A., Krasakopoulou, E. and Nomikou, P., 2018. Coccolithophore community response along a natural CO_2 gradient off Methana (SW Saronikos Gulf, Greece, NE Mediterranean). PLOS ONE, 13(7). https://doi.org/10.1371/journal.pone.0200012
26. Baumann, K.-H., Andruleit, H., Böckel, B., Geisen, M. and Kinkel, H., 2005. The significance of extant coccolithophores as indicators of ocean water masses, surface water temperature, and paleoproductivity: a review. Paläontologische Zeitschrift, 79(1), pp.93–112. https://epic.awi.de/id/eprint/11233/1/Bau2004d.pdf
27. Birger Neuhaus, Robert P. Higgins, Ultrastructure, Biology, and Phylogenetic Relationships of Kinorhyncha, Integrative and Comparative Biology, Volume 42, Issue 3, July 2002, Pages 619–632, https://doi.org/10.1093/icb/42.3.619
28. Todorov, M. and Bankov, N., 2019. *An Atlas of Sphagnum-Dwelling Testate Amoebae in Bulgaria*. Advanced Books. https://doi.org/10.3897/ab.e38685
29. Gąsiorek, P. and Vončina, K., 2019. New Echiniscidae (Heterotardigrada) from Amber Mountain (Northern Madagascar). *Evolutionary Systematics*, 3(1), pp.29–39. https://doi.org/10.3897/evolsyst.3.33580
30. Perry, E.S., Miller, W.R. and Lindsay, S., 2015. Looking at tardigrades in a new light: using epifluorescence to interpret structure. *Journal of Microscopy*, 257(2), pp.117–122. https://doi.org/10.1111/jmi.12190
31. Ernst, F., Fabritius, H.-O., Griesshaber, E., Reisecker, C., Neues, F., Epple, M., Schmahl, W.W., Hild, S. & Ziegler, A., 2020. Functional adaptations in the tergite cuticle

of the desert isopod Hemilepistus reaumuri (Milne-Edwards, 1840). Journal of Structural Biology, 212(1), p.107570. https://doi.org/10.1016/j.jsb.2020.107570
32. Wesener, T. & Schütte, K., 2010. Swarming behaviour and mass occurrences in the world's largest giant pill-millipede species, *Zoosphaerium neptunus*, on Madagascar and its implication for conservation efforts (Diplopoda: Sphaerotheriida). *Madagascar Conservation & Development*, 5(2), pp.89–94. https://doi.org/10.4314/mcd.v5i2.63137
33. Hoffman, D.L., 1989. Settlement and recruitment patterns of a pedunculate barnacle, Pollicipes polymerus Sowerby, off La Jolla, California. Journal of Experimental Marine Biology and Ecology, 125(2), pp.83–98. https://doi.org/10.1016/0022-0981(89)90036-1
34. Mitchell, R.L., Coleman, M., Davies, P., North, L., Pope, E.C., Pleydell-Pearce, C., Harris, W. & Johnston, R., 2019. Macro-to-nanoscale investigation of wall-plate joints in the acorn barnacle Semibalanus balanoides: correlative imaging, biological form and function, and bioinspiration. Journal of The Royal Society Interface, 16(154), p.20190218. https://doi.org/10.1098/rsif.2019.0218
35. Mason, R.J., Rice, S.P., Wood, P.J. & Johnson, M.F., 2019. The zoogeomorphology of case-building caddisfly: quantifying sediment use. Earth Surface Processes and Landforms, 44(12), pp.2510–2525. https://doi.org/10.1002/esp.4670
36. Trice, E., Tyler, J. & Day, J.C., 2004. Description of pleural defensive organs in three species of firefly larvae (Coleoptera: Lampyridae). Zootaxa, 768(1), pp.1–11. https://doi.org/10.11646/zootaxa.768.1.1
37. *Developmental Biology*, 2014. *Dynamics of F-actin prefigure the structure of butterfly wing scales*. *Developmental Biology*, 396(2), pp.227–237. https://doi.org/10.1016/j.ydbio.2014.06.005
38. Schroeder, T.B.H., Houghtaling, J., Wilts, B.D. & Mayer, M., 2018. *It's Not a Bug, It's a Feature: Functional Materials in Insects*. *Advanced Materials*, 30(17), p.1705322. https://doi.org/10.1002/adma.201705322
39. Siddique, R.H., Gomard, G. & Hölscher, H., 2015. *The role of random nanostructures for the omnidirectional anti-reflection properties of the glasswing butterfly*. *Nature Communications*, 6, p.6909. https://doi.org/10.1038/ncomms7909
40. Peterson, A. (1964). Egg Types among Moths of the Noctuidae (Lepidoptera). *The Florida Entomologist*, *47*(2), 71–91. https://doi.org/10.2307/3493280
41. Isoe, J., Koch, L.E., Isoe, Y.E., Rascón Jr., A.A., Brown, H.E., Massani, B.B., Miesfeld, R.L., 2019. Identification and characterization of a mosquito-specific eggshell organizing factor in Aedes aegypti mosquitoes. PLOS Biology, 17(1), e3000068. https://doi.org/10.1371/journal.pbio.3000068
42. Doekele G. Stavenga; Reflections on colourful ommatidia of butterfly eyes. *J Exp Biol* 15 April 2002; 205 (8): 1077–1085. doi: https://doi.org/10.1242/jeb.205.8.1077
43. Gorb, S.N., 2006. Ultrastructure of the neck membrane in dragonflies (Insecta: Odonata). Journal of Zoology, 250(4), pp.479–494. https://doi.org/10.1111/j.1469-7998.2000.tb00791.x
44. Schroeder, T.B.H., Houghtaling, J., Wilts, B.D. & Mayer, M., 2018. It's Not a Bug, It's a Feature: Functional Materials in Insects. Advanced Materials, 30(19), p.1705322. https://doi.org/10.1002/adma.201705322

45. Dinges, G.F., Chockley, A.S., Bockemühl, T., Ito, K., Blanke, A. & Büschges, A., 2020. Location and arrangement of campaniform sensilla in *Drosophila melanogaster*. *Journal of Comparative Neurology*, 528(10), pp.1797–1817. https://doi.org/10.1002/cne.24987
46. Patel, B. H., & Patel, H. K. (1972). New species of *Cyllognatha* Koch and *Thwaitesia* Cambridge (Theridiidae : Araneida) from Gujarat, India. *Oriental Insects*, *6*(3), 293–297. https://doi.org/10.1080/00305316.1972.10434078
47. Lapointe, S.L., Hunter, W.B. & Alessandro, R.T., 2004. Cuticular hydrocarbons on elytra of the Diaprepes root weevil *Diaprepes abbreviatus* (L.) (Coleoptera: Curculionidae). *Agricultural and Forest Entomology*, 6(4), pp.251–257. https://doi.org/10.1111/j.1461-9555.2004.00230.x
48. Hensel, R., Neinhuis, C. & Werner, C., 2016. The springtail cuticle as a blueprint for omniphobic surfaces. *Chemical Society Reviews*, 45(1), pp.323–341. https://doi.org/10.1039/C5CS00438A
49. Hensel, R., Helbig, R., Aland, S., Voigt, A., Neinhuis, C. & Werner, C., 2013. Tunable nano-replication to explore the omniphobic characteristics of springtail skin. *NPG Asia Materials*, 5, p.e37. https://doi.org/10.1038/am.2013.6
50. Abbott, J., 2003. *Corydalus cornutus* (dobsonfly). *Digital Morphology*. Available at: http://www.digimorph.org/specimens/Corydalus_cornutus/whole/ [Accessed 28 August 2025]
51. Combes, S.A., 2010. Materials, structure, and dynamics of insect wings as bioinspiration for MAVs. In: R. Blockley & W. Shyy, eds. *Encyclopedia of Aerospace Engineering*. Vol. 7, Vehicle Design. John Wiley & Sons, pp. 1–10. https://doi.org/10.1002/9780470686652.eae404
52. Sakakibara, A.M., 2014. A new species of *Oeda* (Hemiptera: Membracidae: Stegaspidinae) from Madre de Dios, Peru. *Zoologia (Curitiba)*, 31(6), pp.557–560. https://doi.org/10.1590/S1984-46702014000600004
53. Bodil A Bluhm, Raouf Kilada, William Ambrose, Paul E Renaud, Jan H Sundet, First record of cuticle bands in the stomach ossicles of the red king crab *Paralithodes camtschaticus* (Tilesius, 1815) (Decapoda: Anomura: Lithodidae) from Norway, *Journal of Crustacean Biology*, Volume 39, Issue 6, November 2019, Pages 703–710, https://doi.org/10.1093/jcbiol/ruz064
54. Juan C. Gutiérrez-Marco, Artur A. Sá, Diego C. García-Bellido, Isabel Rábano, Manuel Valério; Giant trilobites and trilobite clusters from the Ordovician of Portugal. *Geology* 2009;; 37 (5): 443–446. doi: https://doi.org/10.1130/G25513A.1
55. Likhitrakarn N, Golovatch SI, Semenyuk I, Efeykin BD, Panha S (2019) Review of the millipede genus *Orthomorpha* Bollman, 1893 (Diplopoda, Polydesmida, Paradoxosomatidae) in Vietnam, with several new records and descriptions of two new species. ZooKeys 898: 121-158. https://doi.org/10.3897/zookeys.898.39265
56. Lowenstam, Heinz A, and Stephen Weiner, 'Arthropoda', *On Biomineralization* (New York, 1989; online edn, Oxford Academic, 12 Nov. 2020), https://doi.org/10.1093/oso/9780195049770.003.0009, accessed 28 Aug. 2025.
57. Clarkson, E.N.K., Levi-Setti, R. & Horváth, G., 2007. The eyes of trilobites: The oldest preserved visual system. *Arthropod Structure & Development*, 35(4), pp.247–259. https://doi.org/10.1016/j.asd.2006.08.002

58. Schoenemann, B., 2021. An overview on trilobite eyes and their functioning. *Arthropod Structure & Development*, 61, p.101032. https://doi.org/10.1016/j.asd.2021.101032
59. Glazier, D.S., Powell, M.G. & Deptola, T.J., 2013. Body-size scaling of metabolic rate in the trilobite *Eldredgeops rana*. *Paleobiology*, 39(1), pp.109–122. https://doi.org/10.1666/0094-8373-39.1.109
60. Zhang, Y., Chen, C. & Qiu, J.-W., 2018. Sexually dimorphic scale worms (Annelida: Polynoidae) from hydrothermal vents in the Okinawa Trough: Two new species and two new sex morphs. *Frontiers in Marine Science*, 5, p.112. https://doi.org/10.3389/fmars.2018.00112
61. Parapar, J., Palomanes, V., Helgason, G.V. & Moreira, J., 2020. Taxonomy and distribution of Pectinariidae (Annelida) from Iceland with a comparative analysis of uncinal morphology. *European Journal of Taxonomy*, 666, pp.1–32. https://doi.org/10.5852/ejt.2020.666
62. Zhang, Y., Chen, C. & Qiu, J.-W., 2013. Sexually dimorphic scale worms (Annelida: Polynoidae) from hydrothermal vents in the Okinawa Trough: Two new species and two new sex morphs. *Frontiers in Marine Science*, 5, p.112. https://doi.org/10.3389/fmars.2018.00112
63. Schwarz, D., Gorb, S.N., Kovalev, A., Konow, N. & Heiss, E., 2020. Not just scratching the surface: distinct radular motion patterns in molluscs. *Biology Open*, 9(10), p.bio055699. https://doi.org/10.1242/bio.055699
64. Reyes-Gómez, A., Ortigosa, D. & Simões, N., 2017. Chitons (Mollusca, Polyplacophora) from Alacranes Reef, Yucatán, Mexico. *ZooKeys*, 665, pp.1–36. https://doi.org/10.3897/zookeys.665.10476
65. Vinther, J., 2005. The Early Cambrian *Halkieria* is a mollusc. *Zoologica Scripta*, 34(1), pp.81–89. https://doi.org/10.1111/j.1463-6409.2005.00177.x
66. Li, J., Wang, C., Wu, Y., Wu, M., Wang, L., Wang, Y., Zang, J., 2012. Crystal structure of Sa239. *Journal of Structural Biology*, 177(3), pp.578–582. https://doi.org/10.1016/j.jsb.2011.12.019
67. Connors, M., Yang, T., Hosny, A. *et al.* Bioinspired design of flexible armor based on chiton scales. *Nat Commun* 10, 5413 (2019). https://doi.org/10.1038/s41467-019-13215-0
68. SuzukiMichio and NagasawaHiromichi. 2013. Mollusk shell structures and their formation mechanism. *Canadian Journal of Zoology*. 91(6): 349-366. https://doi.org/10.1139/cjz-2012-0333
69. Furuhashi, T., Schwarzinger, C., Miksik, I., Smrz, M. & Beran, A., 2009. Molluscan shell evolution with review of shell calcification hypothesis. *Comparative Biochemistry and Physiology Part B: Biochemistry and Molecular Biology*, 154(3), pp.351–371. https://doi.org/10.1016/j.cbpb.2009.07.011
70. Gotliv, B.A., Addadi, L. & Weiner, S., 2003. Mollusk shell acidic proteins: In search of individual functions. *ChemBioChem*, 4(6), pp.522–529. https://doi.org/10.1002/cbic.200200548
71. Peterman, D.J., Mikami, T. & Inoue, S., 2020. The balancing act of *Nipponites mirabilis* (Nostoceratidae, Ammonoidea): Managing hydrostatics throughout a complex ontogeny. *PLOS ONE*, 15(8), e0235180. https://doi.org/10.1371/journal.pone.0235180

72. Ziegler, A. & Zachos, L., 2008. *Echinoneus cyclostomus* (Little Burrowing Urchin). Digital Morphology. Available at: http://www.digimorph.org/specimens/Echinoneus_cyclostomus/
73. Smirnov, A.V. Parallelisms in the evolution of sea cucumbers (Echinodermata: Holothuroidea). *Paleontol. J.* 50, 1610–1625 (2016). https://doi.org/10.1134/S0031030116140082
74. Smirnov, A.V. Parallelisms in the evolution of sea cucumbers (Echinodermata: Holothuroidea). *Paleontol. J.* 50, 1610–1625 (2016). https://doi.org/10.1134/S0031030116140082
75. Tomholt, L., Friesen, L.J., Berdichevsky, D., Fernandes, M.C., Pierre, C., Wood, R.J. & Weaver, J.C., 2020. The structural origins of brittle star arm kinematics: An integrated tomographic, additive manufacturing, and parametric modeling-based approach. Journal of Structural Biology, 211(1), p.107481. https://doi.org/10.1016/j.jsb.2020.107481
76. Wilbur, B., 1995. *Pisaster sp.* (starfish). Digital Morphology. Available at: http://www.digimorph.org/specimens/Pisaster_sp/
77. Blowes, L.M., Egertová, M., Liu, Y., Davis, G.R., Terrill, N.J., Gupta, H.S. & Elphick, M.R., 2017. Body wall structure in the starfish *Asterias rubens*. *Journal of Anatomy*, 231(3), pp.325–341. https://doi.org/10.1111/joa.12646
78. Lewis, R.D., Chambers, C.R. & Peebles, M.W., 1990. Grain morphologies and surface textures of Recent and Pleistocene crinoid ossicles, San Salvador, Bahamas. *PALAIOS*, 5(6), pp.570–576. https://doi.org/10.2307/3514862
79. Daniel A. Janies, Janet R. Voight, Marymegan Daly, Echinoderm Phylogeny Including *Xyloplax*, a Progenetic Asteroid, *Systematic Biology*, Volume 60, Issue 4, July 2011, Pages 420–438, https://doi.org/10.1093/sysbio/syr044
80. Baker, A.N., Rowe, F.W.E. & Clark, H.L., 1986. The morphology, development and taxonomic status of *Xyloplax janetae* (Echinodermata: Concentricycloidea). *Transactions of the Royal Society of New Zealand*, 16(2), pp. 29–38. https://www.jstor.org/stable/36229
81. Hess, H., Ausich, W.I., Brett, C.E. & Simms, M.J., 2000. *Fossil Crinoids*. Cambridge University Press. ISBN: 9780521524407
82. Thet, M.M., Noguchi, M. & Yazaki, I., 2004. Larval and juvenile development of the echinometrid sea urchin *Colobocentrotus mertensii*: Emergence of the peculiar form of spines. *Zoological Science*, 21(3), pp.265–274. https://doi.org/10.2108/zsj.21.265
83. Chen, T.T., 2011. *Microstructure and micromechanics of the sea urchin, Colobocentrotus atratus*. Master's thesis, Massachusetts Institute of Technology. Available at: https://dspace.mit.edu/handle/1721.1/67360
84. de Sousa Rangel, B., Santander-Neto, J., Rici, R.E.G. *et al.* Dental sexual dimorphism and morphology of *Urotrygon microphthalmum* . *Zoomorphology* 135, 367–374 (2016). https://doi.org/10.1007/s00435-016-0312-0
85. Seidel, R., Lyons, K., Blumer, M., Zaslansky, P., Fratzl, P., Weaver, J.C. & Dean, M.N., 2016. Ultrastructural and developmental features of the tessellated endoskeleton of elasmobranchs (sharks and rays). *Journal of Anatomy*, 229(2), pp.232–246. https://doi.org/10.1111/joa.12508

86. Lang, A., Habegger, M.L., Motta, P. (2015). Shark Skin Drag Reduction. In: Bhushan, B. (eds) Encyclopedia of Nanotechnology. Springer, Dordrecht. https://doi.org/10.1007/978-94-007-6178-0_266-2
87. Lauder, G. V., Wainwright, D. K., Domel, A. G., Weaver, J. C., Wen, L., & Bertoldi, K. (2016). Structure, biomimetics, and fluid dynamics of fish skin surfaces. *Physical Review Fluids*, 1(6), 060502. https://doi.org/10.1103/PhysRevFluids.1.060502
88. Clark, B., Chaumel, J., Johanson, Z., Underwood, C., Smith, M.M. & Dean, M.N., 2022. Bricks, trusses and superstructures: Strategies for skeletal reinforcement in batoid fishes (rays and skates). *Frontiers in Cell and Developmental Biology*, 10, p.932341. Available at: https://doi.org/10.3389/fcell.2022.932341
89. White, W.T. & Moore, A.B.M., 2013. Redescription of Aetobatus flagellum (Bloch & Schneider, 1801), an endangered eagle ray (Myliobatoidea: Myliobatidae) from the Indo–West Pacific. *Zootaxa*, 3752(1), pp.199–213. Available at: https://doi.org/10.11646/zootaxa.3752.1.12
90. Spinner, M., Kortmann, M., Traini, C. *et al.* Key role of scale morphology in flatfishes (Pleuronectiformes) in the ability to keep sand. *Sci Rep* 6, 26308 (2016). https://doi.org/10.1038/srep26308
91. Turner, S., Vergoossen, J.M.J. & Williams, R.B., 1995. Early Devonian microvertebrates from Pwll-y-Wrach, Talgarth, South Wales. Geobios, Special Memoir No. 19, pp. 377–382. https://doi.org/10.1016/S0016-6995(95)80142-1
92. Kannan K, Ajith KTT, Zacharia PU, Joshi KK (2017) New Record of Satyrichthys milleri Kawai, 2013 (Peristediidae) From Gulf of Mannar, Bay of Bengal. J Aquac Mar Biol 5(6): 00141 DOI: 10.15406/jamb.2017.05.00141
93. Song, J., Reichert, S., Kallai, I., Gazit, D., Wund, M., Boyce, M.C. & Ortiz, C., 2010. Quantitative microstructural studies of the armor of the marine threespine stickleback (*Gasterosteus aculeatus*). *Journal of Structural Biology*, 171(3), pp.318–331. Available at: https://doi.org/10.1016/j.jsb.2010.05.003
94. Britz, R. & Johnson, G.D., 2002. “Paradox Lost”: Skeletal ontogeny of *Indostomus paradoxus* and its significance for the phylogenetic relationships of Indostomidae (*Teleostei*, *Gasterosteiformes*). *American Museum Novitates*, 3383, pp.1–43. Available at: https://doi.org/10.1206/0003-0082(2002)383
95. Spinner, M., Kortmann, M., Traini, C. *et al.* Key role of scale morphology in flatfishes (Pleuronectiformes) in the ability to keep sand. *Sci Rep* 6, 26308 (2016). https://doi.org/10.1038/srep26308
96. M R Minicozzi, J Perez, D S Kimball, A C Gibb, Scale Thickness Predicts Skin Puncture-Force Resistance in Three Pleuronectiform Fishes, *Integrative Organismal Biology*, Volume 1, Issue 1, 2019, obz005, https://doi.org/10.1093/iob/obz005
97. Spinner, M., Schaber, C.F., Chen, S.-M., Geiger, M., Gorb, S.N. & Rajabi, H., 2019. Mechanical behavior of ctenoid scales: Joint-like structures control the deformability of the scales in the flatfish *Solea solea* (*Pleuronectiformes*). *Acta Biomaterialia*, 92, pp.305–314. Available at: https://doi.org/10.1016/j.actbio.2019.04.019
98. SOHAN L. JAIN, Variability of dermal bones and other parameters in the skull of *Amia calva*, *Zoological Journal of the Linnean Society*, Volume 84, Issue 4, August 1985, Pages 385–395, https://doi.org/10.1111/j.1096-3642.1985.tb01805.x

99. Grande, L., & Bemis, W. E. (1998). A Comprehensive Phylogenetic Study of Amiid Fishes (Amiidae) Based on Comparative Skeletal Anatomy. an Empirical Search for Interconnected Patterns of Natural History. *Journal of Vertebrate Paleontology*, *18*(sup1), 1–696. https://doi.org/10.1080/02724634.1998.10011114
100. Yang, W., Gludovatz, B., Zimmermann, E.A., Bale, H.A., Ritchie, R.O. & Meyers, M.A., 2013. Structure and fracture resistance of alligator gar (*Atractosteus spatula*) armored fish scales. *Acta Biomaterialia*, 9(4), pp.5876–5889. https://doi.org/10.1016/j.actbio.2012.12.026
101. Yang, W., Naleway, S.E., Porter, M.M., Meyers, M.A. & McKittrick, J., 2015. The armored carapace of the boxfish. *Acta Biomaterialia*, 23, pp.1–10. https://doi.org/10.1016/j.actbio.2015.05.024
102. Lowe, A., Summers, A.P., Walter, R.P., Walker, S. & Paig-Tran, E.W.M., 2018. Scale performance and composition in a small Amazonian armored catfish, *Corydoras trilineatus*. *Journal of Experimental Biology*, 221(16), jeb183416. https://doi.org/10.1242/jeb.183416
103. Carr, A., Kemp, A., Tibbetts, I., Truss, R. & Drennan, J., 2006. Microstructure of pharyngeal tooth enameloid in the parrotfish *Scarus rivulatus* (Pisces: Scaridae). *Journal of Microscopy*, 222(3), pp.202–214. https://doi.org/10.1111/j.1365-2818.2006.01526.x
104. Marcus, M.A., Amini, S., Stifler, C.A., Sun, C.-Y., Tamura, N., Bechtel, H.A., Parkinson, D.Y., Barnard, H.S., Zhang, X.X.X., Chua, J.Q.I., Miserez, A. & Gilbert, P.U.P.A., 2017. Parrotfish teeth: stiff biominerals whose microstructure makes them tough and abrasion-resistant to bite stony corals. *ACS Nano*, 11(11), pp.11856–11864. https://doi.org/10.1021/acsnano.7b05044
105. Arratia, G., Schultze, H.-P. & Wilson, M.V.H., 2013. Miniature armored teleosts from the Albian-Cenomanian (Cretaceous) of Mexico. In: G. Arratia, H.-P. Schultze & M.V.H. Wilson, eds. *Mesozoic Fishes 5 – Global Diversity and Evolution*. München: Verlag Dr. F. Pfeil, pp.457–487.
106. Kolmann, M.A., Urban, P. & Summers, A.P., 2018. Structure and function of the armored keel in piranhas, pacus, and their allies. *Anatomical Record*, 301(11), pp.1912–1927. https://doi.org/10.1002/ar.23986
107. Fava, D. & Toledo-Piza, M., 2007. Egg surface structure in the annual fishes *Simpsonichthys* (subgenera *Ophthalmolebias* and *Xenurolebias*) and *Nematolebias* (Teleostei: Cyprinodontiformes: Rivulidae): variability and phylogenetic significance. *Journal of Fish Biology*, 71(3), pp.623–640. https://doi.org/10.1111/j.1095-8649.2007.01572.
108. Thompson, A.W., 2016. *The evolution of chorionic ultrastructure, delayed hatching, and desiccation tolerance in annual killifishes*. PhD thesis, The George Washington University. ProQuest Dissertations & Theses, 10165658
109. Dezfuli, B.S., Giari, L., Castaldelli, G. *et al.* Temporal and spatial changes in the composition and structure of helminth component communities in European eels *Anguilla anguilla* in an Adriatic coastal lagoon and some freshwaters in Italy. *Parasitol Res* 113, 113–120 (2014). https://doi.org/10.1007/s00436-013-3633-7
110. Britz, R. & Johnson, G.D., 2002. "Paradox Lost": Skeletal ontogeny of *Indostomus paradoxus* and its significance for the phylogenetic relationships of

Indostomidae (Teleostei, Gasterosteiformes). *American Museum Novitates*, 3383, pp.1–43. https://doi.org/10.1206/0003-0082(2002)383

111. Song, J., Reichert, S., Kallai, I., Gazit, D., Wund, M., Boyce, M.C. & Ortiz, C., 2010. Quantitative microstructural studies of the armor of the marine threespine stickleback (*Gasterosteus aculeatus*). *Journal of Structural Biology*, 171(3), pp.318–331. https://doi.org/10.1016/j.jsb.2010.05.003
112. Ruibal, R., & Shoemaker, V. (1984). Osteoderms in Anurans. *Journal of Herpetology*, *18*(3), 313–328. https://doi.org/10.2307/1564085
113. Federle, W., Barnes, W.J.P., Baumgartner, W., Drechsler, P. & Smith, J.M., 2006. Wet but not slippery: boundary friction in tree frog adhesive toe pads. *Journal of the Royal Society Interface*, 3(7), pp.689–697. https://doi.org/10.1098/rsif.2006.0135
114. Chang, C., Wu, P., Baker, R.E., Maini, P.K., Alibardi, L. & Chuong, C.-M., 2009. Reptile scale paradigm: Evo-Devo, pattern formation and regeneration. *International Journal of Developmental Biology*, 53(5-6), pp.813–826. https://doi.org/10.1387/ijdb.072556cc
115. Scherz, M.D., Daza, J.D., Köhler, J., Vences, M. & Glaw, F., 2017. Off the scale: a new species of fish-scale gecko (*Geckolepis*) with exceptionally large scales. *PeerJ*, 5, e2955. https://doi.org/10.7717/peerj.2955
116. Ranajay Ghosh, Hamid Ebrahimi, Ashkan Vaziri; Contact kinematics of biomimetic scales. *Appl. Phys. Lett.* 8 December 2014; 105 (23): 233701. https://doi.org/10.1063/1.4903160
117. Vickaryous, M.K. & Sire, J.-Y., 2009. The integumentary skeleton of tetrapods: origin, evolution, and development. *Journal of Anatomy*, 214(4), pp.441–464. https://doi.org/10.1111/j.1469-7580.2008.01043.x
118. Laver, R.J., Morales, C.H., Heinicke, M.P., Gamble, T., Longoria, K., Bauer, A.M. & Daza, J.D., 2019. The development of cephalic armor in the tokay gecko (Squamata: Gekkonidae: *Gekko gecko*). *Journal of Morphology*, 280(12), pp.1775–1792. https://doi.org/10.1002/jmor.21092
119. Broeckhoven, C., El Adak, Y., Hui, C., Van Damme, R. & Stankowich, T., 2018. On dangerous ground: the evolution of body armour in cordyline lizards. *Proceedings of the Royal Society B: Biological Sciences*, 285(1880), 20180513. https://doi.org/10.1098/rspb.2018.0513
120. Broeckhoven, C., du Plessis, A., Le Roux, S.G., Le Fras N. Mouton, P. & Hui, C., 2016. In vivo micro-CT scanning: Studying reptiles and amphibians from the inside out. Methods Blog. Available at: https://methodsblog.com/2016/11/11/in-vivo-micro-ct-scanning/
121.
122. Baumgartner, W., Saxe, F., Weth, A. *et al.* The sandfish’s skin: Morphology, chemistry and reconstruction. *J Bionic Eng* 4, 1–9 (2007). https://doi.org/10.1016/S1672-6529(07)60006-7
123. Krauss, S., Monsonego-Ornan, E., Zelzer, E., Fratzl, P. & Shahar, R., 2009. Mechanical function of a complex three-dimensional suture joining the bony elements in the shell of the red-eared slider turtle. *Advanced Materials*, 21(6), pp.567–572. https://doi.org/10.1002/adma.200801256

124. Wang, B., Yang, W., McKittrick, J. & Meyers, M.A., 2016. Keratin: Structure, mechanical properties, occurrence in biological organisms, and efforts at bioinspiration. *Progress in Materials Science*, 76, pp.229–318. https://doi.org/10.1016/j.pmatsci.2015.06.002
125. Chen, M., Hu, N., Zhou, C., Lin, X., Xie, H. & He, Q., 2019. The hierarchical structure and mechanical performance of a natural nanocomposite material: The turtle shell. *Materials & Design*, 178, 107868. https://doi.org/10.1016/j.matdes.2019.107868
126. Achrai, B., Bar-On, B. & Wagner, H.D., 2015. Biological armors under impact—effect of keratin coating, and synthetic bio-inspired analogues. *Bioinspiration & Biomimetics*, 10(1), 016009. https://doi.org/10.1088/1748-3190/10/1/016009
127. Lee N, Horstemeyer MF, Rhee H, Nabors B, Liao J, Williams LN. 2014 Hierarchical multiscale structure–property relationships of the red-bellied woodpecker (Melanerpes arolinus) beak. J. R. Soc. Interface 11: 20140274. http://dx.doi.org/10.1098/rsif.2014.0274
128. Scheyer, T. M., & Sander, P. M. (2004). Histology of ankylosaur osteoderms: implications for systematics and function. *Journal of Vertebrate Paleontology*, *24*(4), 874–893. https://doi.org/10.1671/0272-4634(2004)024[0874:HOAOIF]2.0.CO;2
129. Ford, T., 2002. A new look at the armor of *Ankylosaurus*, just how did it look? *In: Dinosaur enthusiasts* [online]. June 2002
130. Hayashi, S., Carpenter, K., Scheyer, T.M., Watabe, M. & Suzuki, D., 2010. Function and evolution of ankylosaur dermal armor. *Acta Palaeontologica Polonica*, 55(2), pp.213–228. http://dx.doi.org/10.4202/app.2009.0103
131. Ford, T., 2000. A review of ankylosaur osteoderms from New Mexico and a preliminary review of ankylosaur armor. *In: Dinosaur enthusiasts* [online]. January 2000.
132. Riedel, J., Böhme, W., Bleckmann, H. & Spinner, M., 2014. Microornamentation of leaf chameleons (Chamaeleonidae: *Brookesia*, *Rhampholeon*, and *Rieppeleon*)—with comments on the evolution of microstructures in the Chamaeleonidae. *Journal of Morphology*, 275(11), pp.1319–1338. https://doi.org/10.1002/jmor.20330
133. Iacoviello, F., Kirby, A.C., Javanmardi, Y., Moeendarbary, E., Shabanli, M., Tsolaki, E., Sharp, A.C., Hayes, M.J., Keevend, K., Li, J.-H., Brett, D.J.L., Shearing, P.R., Olivo, A., Herrmann, I.K., Evans, S.E., Moazen, M. and Bertazzo, S., 2020. The multiscale hierarchical structure of Heloderma suspectum osteoderms and their mechanical properties. *Acta Biomaterialia*, 107, pp.194–203. https://doi.org/10.1016/j.actbio.2020.02.029
134. Maisano, J.A., LaDuc, T.J., Bell, C.J. and Barber, D., 2019. The cephalic osteoderms of Varanus komodoensis as revealed by high-resolution X-ray computed tomography. *The Anatomical Record*, 302(10), pp.1675–1680. https://doi.org/10.1002/ar.24197 .
135. Martins, A.F., Bennett, N.C., Clavel, S. *et al.* Locally-curved geometry generates bending cracks in the African elephant skin. *Nat Commun* 9, 3865 (2018). https://doi.org/10.1038/s41467-018-06257-3
136. Lillywhite, H.B. and Stein, B.R., 1987. Surface sculpturing and water retention of elephant skin. *Journal of Zoology*, 211(4), pp.727–734. https://doi.org/10.1111/j.1469-7998.1987.tb04483.x

137. J. M. Mahoney and H. I. Rosenberg. 1981. Anatomy of the tail in the beaver (*Castor canadensis*). *Canadian Journal of Zoology*. 59(3): 390-399. https://doi.org/10.1139/z81-057
138. Aleksiuk, M., 1970. The function of the tail as a fat storage depot in the beaver (*Castor canadensis*). *Journal of Mammalogy*, 51(1), pp.145–148. https://doi.org/10.2307/1378541
139. Carlson, M. and Welker, W.I., 1976. Some morphological, physiological and behavioral specializations in North American beavers (*Castor canadensis*). *Brain, Behavior and Evolution*, 13(4), pp.302–326. https://doi.org/10.1159/000123818
140. Chen, I.H., Kiang, J.H., Correa, V., Lopez, M.I., Chen, P.Y., McKittrick, J. and Meyers, M.A., 2011. Armadillo armor: Mechanical testing and micro-structural evaluation. *Journal of the Mechanical Behavior of Biomedical Materials*, 4(5), pp.713–722. https://doi.org/10.1016/j.jmbbm.2010.12.013
141. Zurita, A.E., Soibelzon, L.H., Soibelzon, E., Gasparini, G.M., Cenizo, M.M. and Arzani, H., 2010. Accessory protection structures in Glyptodon Owen (Xenarthra, Cingulata, Glyptodontidae). *Annales de Paléontologie*, 96(1), pp.1–11. https://doi.org/10.1016/j.annpal.2010.01.001 .
142. Wang, B., Yang, W., Sherman, V.R. and Meyers, M.A., 2016. Pangolin armor: Overlapping, structure, and mechanical properties of the keratinous scales. *Acta Biomaterialia*, 42, pp.193–202. https://doi.org/10.1016/j.actbio.2016.06.018
143. Liu, Z.Q., Jiao, D., Weng, Z.Y. and Zhang, Z.F., 2016. Structure and mechanical behaviors of protective armored pangolin scales and effects of hydration and orientation. *Journal of the Mechanical Behavior of Biomedical Materials*, 56, pp.165–174. https://doi.org/10.1016/j.jmbbm.2015.11.013
144. Maden, M., Polvadore, T., Polanco, A., Barbazuk, W.B. and Stanley, E., 2023. Osteoderms in a mammal, the spiny mouse Acomys, and the independent evolution of dermal armor. *iScience*, 26(6), p.106779. https://doi.org/10.1016/j.isci.2023.106779
